# Regulating lattice oxygen participation through Ta-enabled high-entropy engineering for durable acidic oxygen evolution

**DOI:** 10.1039/d6sc05092a

**Published:** 2026-07-31

**Authors:** Yingdi Qiu, Yinze Yang, Gemaming Zhang, Juan Li, Xiaochen Wang, Yibo Dang, Lili Zhang, Huishan Shang, Guoli Zhou, Ning Zhang

**Affiliations:** a State Key Laboratory of Critical Metals Beneficiation, Metallurgy and Purification, Zhengzhou University Zhengzhou 450001 P. R. China nzhang@zzu.edu.cn zglcumt@126.com; b School of Chemical Engineering, Zhengzhou University Zhengzhou 450001 P. R. China shanghs@zzu.edu.cn

## Abstract

Developing acidic oxygen evolution reaction (OER) catalysts with both high activity and high durability is essential for implementing a proton exchange membrane water electrolyzer (PEMWE) in sustainable hydrogen production. However, achieving efficient lattice oxygen activation while maintaining structural robustness during acidic OER remains a fundamental challenge due to the intrinsic activity–stability trade-off associated with lattice oxygen participation. Herein, we report a robust RuMnCoNiTa–O high-entropy oxide catalyst enabled by Ta-mediated electronic regulation and entropy-stabilized local coordination engineering. The optimized catalyst delivers a low overpotential of 196 mV at 10 mA cm^−2^ together with outstanding durability exceeding 500 h under acidic conditions, substantially outperforming commercial RuO_2_. *Operando* spectroscopic investigations combined with Ru dissolution analysis reveal that the Ta-enabled high-entropy structure promotes lattice oxygen participation while mitigating structural degradation and Ru dissolution during the OER. When employed as the anode catalyst in a membrane electrode assembly (MEA), it delivers stable operation for over 280 h at 200 mA cm^−2^, verifying its practical potential for hydrogen production. This work demonstrates that rational regulation of the local coordination and electronic environment can decouple lattice oxygen activation from catalyst instability, providing an effective strategy for overcoming the activity–stability trade-off in acidic OER catalysis.

## Abstract 2

The high-entropy effect stabilizes the oxide lattice, while Ta^5+^ electronic modulation regulates Ru–O coordination, promotes lattice oxygen participation, and suppresses Ru over-oxidation and leaching, enabling active and durable acidic OER.

## Graphical abstract

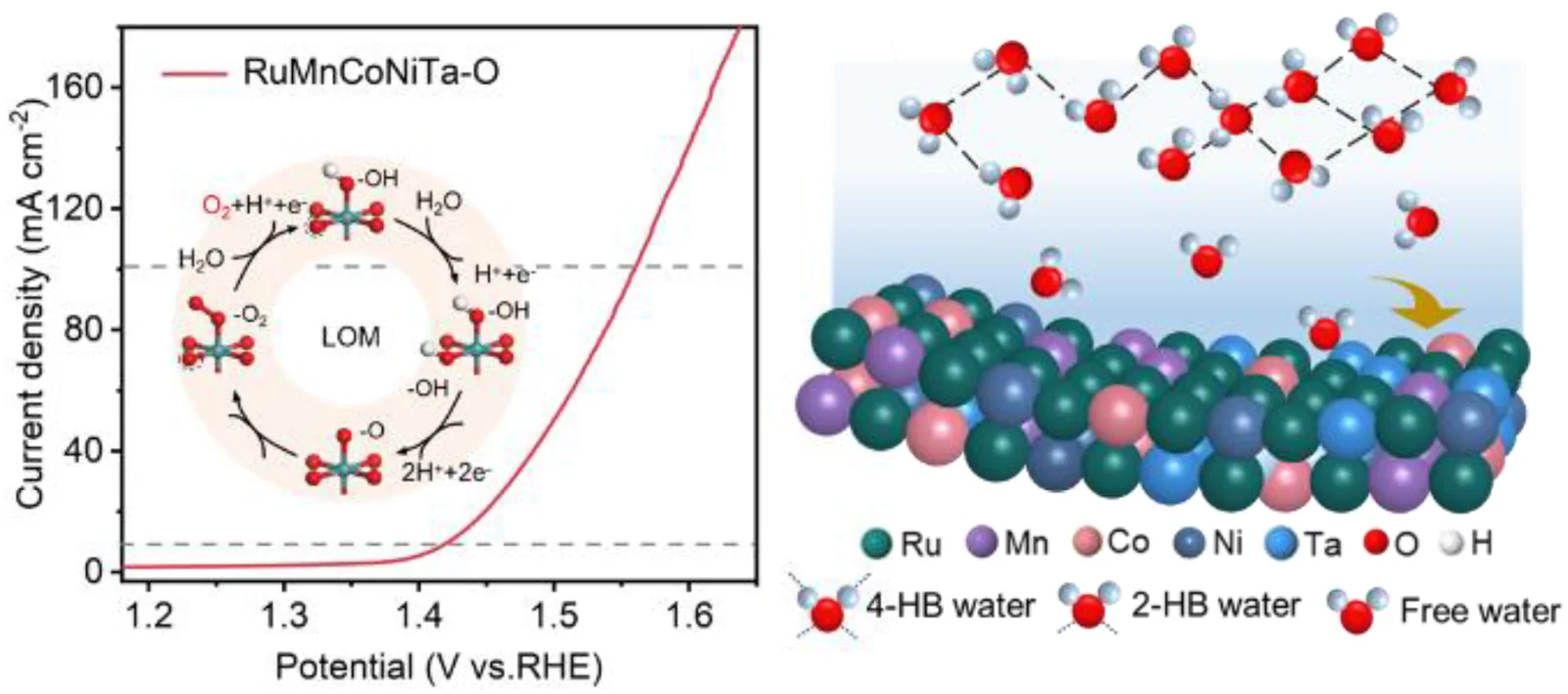

## 1. Introduction

Proton exchange membrane water electrolyzers (PEMWEs) have emerged as a promising technology for sustainable hydrogen production due to their high energy efficiency and rapid response.^1–3^ However, the anodic oxygen evolution reaction (OER) suffers from intrinsically sluggish kinetics, imposing stringent requirements on catalyst activity and durability under harsh acidic and highly oxidative conditions.^4,5^ Ru-based oxides are widely recognized as promising catalysts for acidic OER owing to their high intrinsic activity and relatively lower cost compared to Ir-based counterparts.^6–8^ However, under practical operating conditions, Ru species are thermodynamically prone to over-oxidation to soluble high-valence states (*e.g.*, Ru^6+^ and RuO_4_^2−^),^9–11^ thereby severely limiting their long-term stability at industrially relevant current densities.

Recent studies have revealed that the catalytic performance of Ru-based oxides is closely associated with the underlying reaction pathways. In contrast to the conventional adsorbate evolution mechanism (AEM) followed by crystalline RuO_2_, the lattice oxygen mechanism (LOM), in which lattice oxygen directly participates in O–O bond formation, has emerged as a promising alternative pathway.^12^ This unique feature enables the reaction to bypass the scaling relationship constraints of critical adsorbed intermediates (*O and *OOH) and lowers the overall reaction energy barrier, thereby enhancing catalytic activity.^13^ However, spontaneous lattice oxygen activation in Ru-based oxides often induces uncontrolled oxygen vacancy accumulation and local lattice distortion, which accelerates structural degradation under acidic conditions.^14–16^ This instability is further exacerbated by the strong metal–oxygen covalency of Ru–O bonds, which promotes lattice oxygen participation at the expense of lattice stability. Consequently, maintaining structural integrity under LOM-involved conditions remains a critical challenge.^17^

High-entropy oxides (HEOs), formed by incorporating multiple metallic elements into a single-phase solid solution, provide diverse local coordination environments and strong synergistic interactions.^18–20^ These features enable precise regulation of the electronic structure and optimization of metal–oxygen bonding, thereby creating favorable conditions for LOM activation. Furthermore, the incorporation of cations with different ionic radii induces pronounced lattice distortion and heterogeneous strain fields, which enhance structural robustness and generate a complex energy landscape for atomic migration.^21–23^ As a result, atomic diffusion is effectively suppressed, mitigating metal dissolution and structural degradation under acidic OER conditions. Among various elements, tantalum (Ta) is particularly attractive due to its high valence state, strong Ta–O bonding, and excellent corrosion resistance in acidic media. The incorporation of Ta into HEOs not only enables precise modulation of the Ru electronic environment but also stabilizes oxygen vacancies and lattice oxygen species, thereby suppressing excessive Ru over-oxidation and dissolution.^24,25^ Lee *et al.* recently reported a high-entropy doping (HED) strategy employing inert d^0^ cations (Ti^4+^, Zr^4+^, Nb^5+^, Hf^4+^, Ta^5+^, and W^6+^) to suppress Ru leaching *via* heterogeneous bonding and multiaxial lattice strain.^26^ This approach proceeds through the adsorbate evolution mechanism (AEM), however, and fails to break the scaling relations governing OER intermediates. We address this limitation with a single-phase quintuple RuMnCoNiTa–O high-entropy oxide. Instead of inert stabilising dopants, our material unites catalytically active Mn, Co, and Ni alongside Ru and Ta to activate the lattice oxygen-mediated mechanism (LOM). Consequently, the Ta-enabled high-entropy strategy offers a promising route to achieve stabilized lattice oxygen activation while maintaining structural integrity during acidic OER.

Herein, we demonstrate that stabilizing lattice oxygen activation through a Ta-enabled high-entropy strategy enables efficient acidic OER catalysis with enhanced durability in Ru-based oxides. The optimized RuMnCoNiTa–O catalyst exhibits a low overpotential of 196 mV at 10 mA cm^−2^ and remarkable durability exceeding 500 h, significantly outperforming commercial RuO_2_. When integrated as the anode in a membrane electrode assembly (MEA), the catalyst maintains stable operation for over 280 h at 200 mA cm^−2^, highlighting its practical applicability. *Operando* characterization combined with Ru dissolution analysis reveals that the Ta-enabled high-entropy structure regulates lattice oxygen participation and suppresses catalyst degradation under acidic conditions. These findings demonstrate that tuning the local coordination environment can decouple lattice oxygen activation from structural instability, offering a viable strategy to break the activity–stability trade-off in acidic OER catalysis.

## 2. Experimental

### 2.1 Preparation of electrocatalysts

Ruthenium(iii) chloride anhydrous (RuCl_3_), manganese(iv) chloride tetrahydrate (MnCl_4_·4H_2_O), cobalt(ii) chloride hexahydrate (CoCl_2_·6H_2_O), nickel(ii) chloride hexahydrate (NiCl_2_·6H_2_O), tantalum(v) chloride anhydrous (TaCl_5_), and sodium nitrate (NaNO_3_) were used as the starting materials to prepare RuMnCoNiTa–O high-entropy oxides by the Adams fusion method, with the molar ratio of each metal ion precursor fixed at 6 : 1 : 1 : 1 : 1. The above metal ion precursors and excess sodium nitrate (2 g) were accurately weighed and added to 10 mL of a mixed solvent of water and ethanol (volume ratio v/v = 1 : 3). The mixed system was continuously stirred at a constant temperature of 80 °C until all raw materials were completely dissolved, yielding a homogeneous and transparent metal salt solution. The obtained metal salt solution was subjected to solvent evaporation treatment. After the solvent was completely volatilized, the remaining solid mixture was fully ground to ensure uniform mixing of the materials and particle refinement. The uniformly ground powder was placed in a tube furnace, heated to 350 °C at a heating rate of 10 °C min^−1^, and calcined at this temperature for 2 h to complete the heat treatment. After the heat treatment, the sample was subjected to rapid cooling treatment, followed by repeated washing with 0.5 mol L^−1^ dilute H_2_SO_4_ and deionized water in sequence to remove residual impurities and soluble salts in the sample. Finally, the target RuMnCoNiTa–O high-entropy oxide catalyst powder was obtained by freeze-drying. To eliminate synthetic variability and precisely isolate high-entropy effects, all reference catalysts were fabricated *via* the same protocol and identical conditions as the quinary RuMnCoNiTa–O catalyst, with only metal salt molar ratios adjusted.

## 3. Results and discussion

### 3.1 Synthesis and structural characterization

RuMnCoNiTa–O high-entropy oxide (HEO) was successfully synthesized *via* a modified Adams fusion strategy, forming an entropy-stabilized single-phase rutile structure. Among the constituent elements, Ru species serve as the primary catalytic centers for acidic OER. The incorporation of Mn^3+^ (0.65 Å), Co^3+^ (0.61 Å), and Ni^3+^ (0.60 Å), possessing ionic radii comparable to Ru^4+^ (0.62 Å), facilitates the formation of a homogeneous solid solution within the rutile lattice. In addition to contributing auxiliary OER activity, these transition-metal species induce local lattice distortion and electronic structure modulation around Ru active sites. In particular, Ta^5+^, featuring robust Ta–O bonding and high bond dissociation energy, effectively reinforces lattice stability and suppresses Ru over-oxidation and dissolution under harsh acidic OER conditions.^27^ The RuMnCoNiTa–O catalyst was synthesized with a Ru : Mn : Co : Ni : Ta atomic ratio of 6 : 1 : 1 : 1 : 1, as quantified by ICP-OES (Fig. S1). Mn, Co, Ni and Ta adopt an approximately equimolar distribution, while Ru is maintained at a higher concentration to function as the structural and catalytically active backbone. The entropy-stabilized MnCoNiTa–O matrix rigidifies the lattice, effectively suppressing the rapid stability degradation inherent to conventional Ru-lean electrocatalysts. In low-Ru systems, the absence of a continuous, robust active framework typically leads to severe performance decay under harsh anodic polarization. This drawback is effectively resolved in our catalyst design, which integrates a Ru-dominant rutile backbone with an entropy-stabilized multimetallic oxide matrix.

The morphological characteristics of RuMnCoNiTa–O were characterized by transmission electron microscopy (TEM) and scanning electron microscopy (SEM), and the acquired images reveal that the material consists of uniformly sized nanoparticles with an average diameter of ∼2.63 nm (Fig. 1a and S2) with no obvious agglomeration. Aberration-corrected high-angle annular dark-field scanning transmission electron microscopy (AC-HAADF-STEM, Fig. 1b) reveals the atomically resolved lattice of the high-entropy oxide. Distinct lattice fringes with an interplanar spacing of ∼0.32 nm are clearly observed, which can be indexed to the (110) plane of rutile RuO_2_, confirming the preservation of the rutile crystalline framework after multi-cation incorporation. Line intensity profiles collected along two paths (line a and line b, Fig. 1c) display pronounced fluctuations in atomic-column intensity, reflecting the random occupation of possible elements within the lattice and suggesting the formation of a homogeneous high-entropy solid solution without obvious elemental segregation or aggregation.^28^

**Fig. 1:**
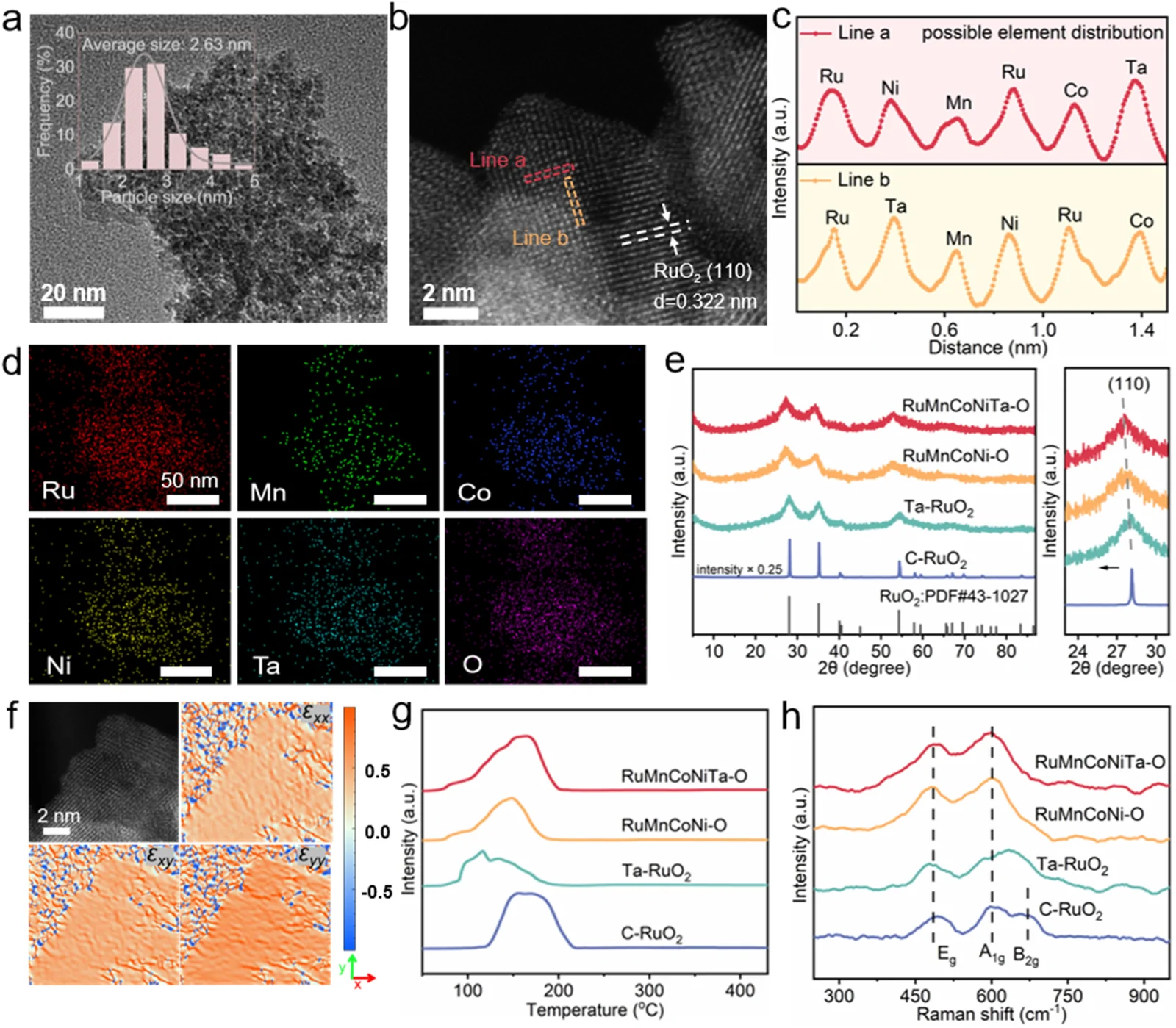
Structural and compositional characterization of RuMnCoNiTa–O and reference catalysts. (a) TEM image of RuMnCoNiTa–O. (b) Aberration-corrected HAADF-STEM image of RuMnCoNiTa–O. (c) Line-scanning intensity profiles collected along atomic columns and the possible elemental distribution within the lattice. (d) STEM-EDS elemental mapping and corresponding elemental distributions of RuMnCoNiTa–O. (e) XRD patterns and enlarged diffraction profiles of the catalysts. (f) GPA images for *ε*_*xx*_, *ε*_*xy*_, and *ε*_*yy*_ strain mapping. (g) H_2_-TPR profiles of the catalysts. (h) Raman spectra of the catalysts.

Energy-dispersive X-ray spectroscopy (EDS) elemental maps (Fig. 1d) reveal the uniform distribution of Ru, Mn, Co, Ni, Ta, and O throughout the nanoparticle without obvious elemental segregation, further confirming the successful formation of a homogeneous single-phase high-entropy oxide. The reference sample RuMnCoNi–O exhibits morphological features similar to those of RuMnCoNiTa–O (Fig. S3 and S4). In addition, the Brunauer–Emmett–Teller (BET) measurements show that RuMnCoNiTa–O possesses a larger specific surface area (177.4 m^2^ g^−1^) than RuMnCoNi–O (96.57 m^2^ g^−1^) (Fig. S5), suggesting a more porous nanostructure with increased exposure of active sites.

Furthermore, powder X-ray diffraction (XRD) was employed to confirm the crystal structure and all diffraction peaks of RuMnCoNiTa–O can be indexed to the rutile RuO_2_ phase (JCPDS no. 43-1027) with no impurity peaks (Fig. 1e). Compared with commercial RuO_2_ (C–RuO_2_), the enlarged view of the (110) diffraction peak exhibits a slight shift toward lower angles, indicating lattice expansion induced by the incorporation of heteroatoms with different ionic radii. The absence of secondary-phase diffraction peaks further supports the formation of a homogeneous high-entropy solid solution. To further probe the local lattice distortion induced by multi-cation incorporation, geometric phase analysis (GPA) was performed on the AC-HAADF-STEM images (Fig. 1f). The corresponding strain maps, including *ε*_*xx*_, *ε*_*yy*_, and shear strain *ε*_*xy*_ components, reveal pronounced lattice strain distributed throughout the RuMnCoNiTa–O nanostructure. Notably, the *ε*_*yy*_ component reaches values of approximately 1%, providing direct evidence of significant local lattice distortion within the rutile structure.^29^ Such entropy-induced lattice strain is expected to generate a sluggish diffusion effect, which increases the energy barrier for cation migration and dissolution, thereby stabilizing the lattice structure and enhancing the acidic OER durability of RuMnCoNiTa–O.

Hydrogen temperature-programmed reduction (H_2_-TPR) profiles (Fig. 1g) disclose the metal–oxygen bond strength and reducibility of the catalysts. C–RuO_2_ exhibits a reduction peak at a relatively low temperature, corresponding to the reduction of Ru^4+^ species to metallic Ru^0^. In contrast, the quinary high-entropy oxide RuMnCoNiTa–O displays a reduction peak centered at approximately 170 °C, which is slightly higher than that of RuMnCoNi–O and comparable to that of C–RuO_2_. The shifted reduction behavior suggests that the incorporation of Ta modulates the Ru–O interaction within the entropy-stabilized lattice,^30^ leading to optimized metal–oxygen covalency and enhanced structural robustness under acidic OER conditions. Such a balanced Ru–O interaction is expected to facilitate lattice oxygen participation during the OER while maintaining catalyst stability. Notably, Ta–RuO_2_ shows a bimodal reduction profile with an overall reduction onset temperature lower than that of C–RuO_2_, indicating that single Ta doping alone fails to effectively strengthen the Ru–O bonds. Raman spectroscopy (Fig. 1h), which is highly sensitive to local lattice symmetry and metal–oxygen coordination environments, was further conducted to investigate the structural characteristics of the catalysts. C–RuO_2_ exhibits three characteristic vibrational modes of the rutile structure located at approximately 450 cm^−1^ (E_g_), 610 cm^−1^ (A_1g_), and 710 cm^−1^ (B_2g_). After the incorporation of multiple heteroatoms, the B_2g_ vibrational mode becomes significantly weakened, accompanied by overall peak broadening, indicating pronounced local lattice distortion and symmetry breaking induced by multication incorporation.^31^ These results further support the successful formation of entropy-stabilized solid-solution structures in RuMnCoNi–O and RuMnCoNiTa–O, consistent with the XRD analysis.

### 3.2 Characterization of electronic and local structures

The valence state of Ru active centers and their local coordination environment are strongly influenced by electronic redistribution among the multiple components, which plays a critical role in determining the electrocatalytic performance.^32^ X-ray photoelectron spectroscopy (XPS) was employed to investigate the surface chemical states and electronic structures of the catalysts. The results confirm that Ru species in both RuMnCoNiTa–O and RuMnCoNi–O exist in oxidized states. Notably, the Ru 3p_3/2_ peak of RuMnCoNiTa–O (Fig. 2a) exhibits an evident positive shift relative to that of RuMnCoNi–O, indicating decreased electron density around the Ru sites. This result reveals substantial electronic redistribution and strong intermetallic interactions within the high-entropy lattice. The altered Ru environment is primarily attributed to the incorporation of high-valence Ta^5+^, which induces charge transfer among neighboring metal species and modulates the local Ru–O electronic structure. Meanwhile, slight negative shifts observed in the Co, Mn and Ni 2p spectra (Fig. 2b and S6) further indicate the presence of synergistic electronic coupling in RuMnCoNiTa–O. Such entropy-induced electronic interactions effectively regulate the surface electronic environment and metal–oxygen covalency of Ru active sites, thereby providing a favorable electronic basis for enhanced acidic OER kinetics and structural stability.

**Fig. 2:**
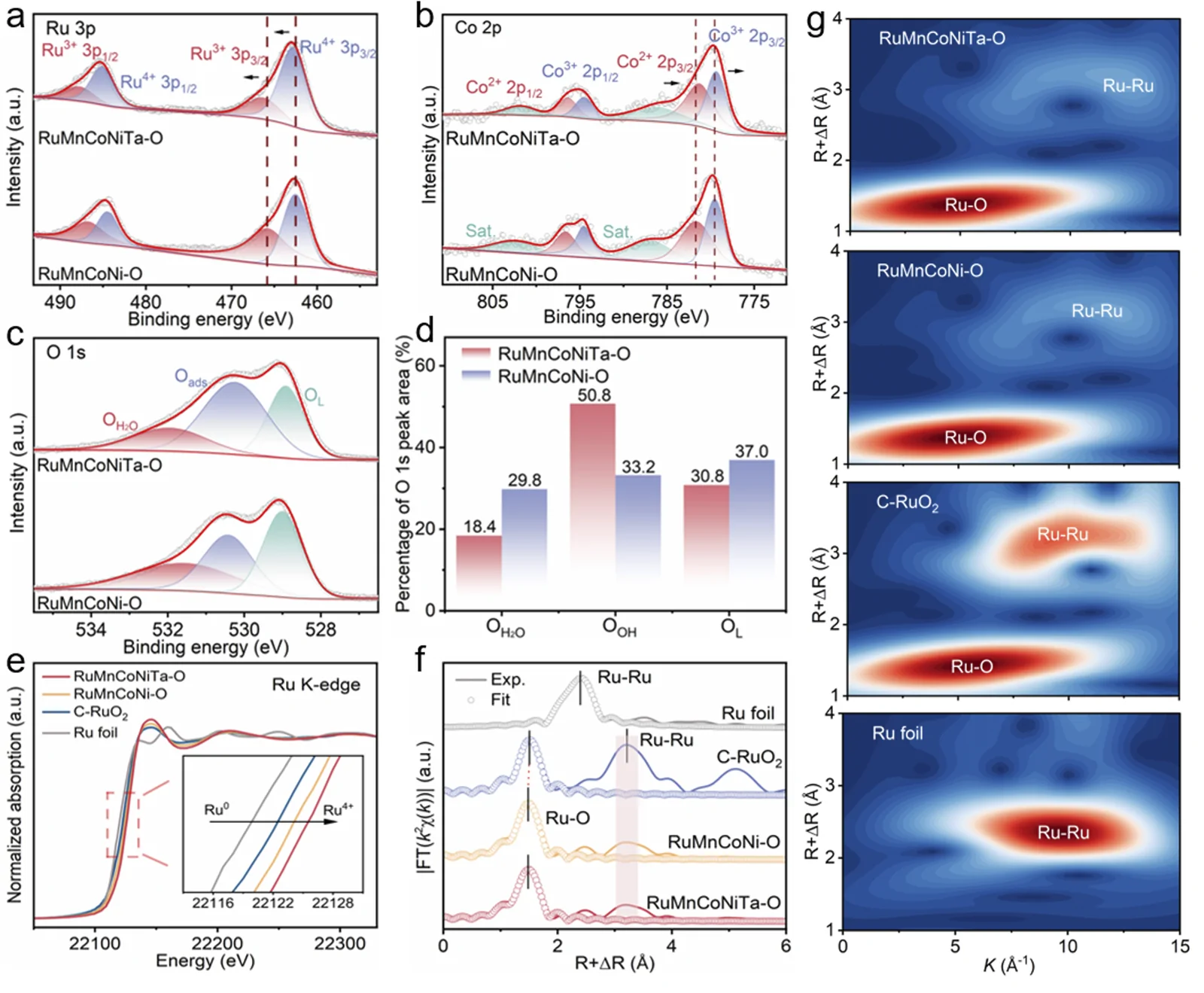
Electronic structure and local coordination environment analyses of catalysts. High-resolution XPS of (a) Ru 3p, (b) Co 2p, and (c) O 1s for RuMnCoNi–O and RuMnCoNiTa–O. (d) Relative area percentages of deconvoluted O 1s components. (e) Ru K-edge XANES of the catalysts. (f) FT-EXAFS of the catalysts at the Ru K-edge. (g) WT-EXAFS contour plots of the catalysts.

The O 1s XPS spectra and corresponding peak-area analysis (Fig. 2c and d) reveal different surface oxygen environments in RuMnCoNiTa–O and RuMnCoNi–O. The deconvoluted O 1s spectra can be assigned to three characteristic oxygen species,^33^ including lattice oxygen (O_L_, 528.9 eV), adsorbed oxygen species (O_ads_, 530.3 eV), and adsorbed water (O_H_2_O_, 532.0 eV). Notably, RuMnCoNiTa–O exhibits a significantly higher proportion of O_ads_ species (48.6%) than RuMnCoNi–O (32.9%). The enriched surface oxygen species suggest increased surface oxygen defects and facilitate oxygen activation and lattice oxygen participation during acidic OER, thereby favoring the LOM pathway.

To further elucidate the local electronic structure and coordination environment of the RuMnCoNiTa–O nanocatalyst, X-ray absorption spectroscopy (XAS) measurements were conducted to probe the valence state and local Ru coordination structure in C–RuO_2_, RuMnCoNi–O, and RuMnCoNiTa–O. As shown in the Ru K-edge XANES spectra (Fig. 2e), the absorption edges of RuMnCoNi–O and RuMnCoNiTa–O shift toward higher energies relative to C–RuO_2_, indicating an increased average oxidation state of Ru species, consistent with the XPS analysis. The elevated oxidation state of Ru can enhance the electrophilicity of Ru active centers, thereby facilitating the nucleophilic attack of water molecules and promoting water dissociation kinetics during acidic OER,^34^ which is beneficial for lattice oxygen-involved reaction pathways.

Fourier-transformed X-ray absorption fine structure (FT-EXAFS) spectra (Fig. 2f) further reveal distinct local coordination characteristics. Metallic Ru foil exhibits a typical Ru–Ru coordination peak, while C–RuO_2_ displays both Ru–O (∼1.5 Å) and Ru–Ru (∼3.2 Å) coordination signals associated with the rutile structure. In contrast, the Ru–Ru coordination peak in RuMnCoNi–O and RuMnCoNiTa–O becomes significantly attenuated, indicating the disruption of long-range Ru ordering induced by high-entropy incorporation. Meanwhile, the slightly shortened Ru–O bond distance observed in RuMnCoNi–O and RuMnCoNiTa–O suggests strengthened metal–oxygen covalency and enhanced local structural distortion. And such local coordination reconstruction is beneficial for charge redistribution and structural stabilization under harsh acidic OER conditions.^35^ In addition, the reduced EXAFS peak intensity and the fitting data (Table S1) indicate decreased Ru coordination numbers and increased structural disorder in RuMnCoNiTa–O, which contribute to the generation of coordinatively unsaturated active sites and enhance the activation of lattice oxygen. Wavelet transform EXAFS (WT-EXAFS) contour plots (Fig. 2g) further visualize the coordination evolution in both *R* and *k* spaces. And the characteristic Ru–Ru scattering signal is largely suppressed in RuMnCoNi–O and RuMnCoNiTa–O, while the Ru–O coordination signal remains dominant. This result further confirms the disruption of long-range Ru–Ru ordering and supports the formation of the homogeneous high-entropy RuMnCoNiTa–O.^36^

### 3.3 Evaluation of electrocatalytic performance

The electrocatalytic OER performance was evaluated in a standard three-electrode configuration using 0.5 M H_2_SO_4_ as the electrolyte. As shown in the linear sweep voltammetry (LSV) curves in Fig. 3a, RuMnCoNiTa–O exhibits remarkably enhanced acidic OER activity compared with the reference catalysts. Specifically, RuMnCoNiTa–O requires overpotentials of only 196 and 323 mV to achieve current densities of 10 and 100 mA cm^−2^, respectively (Fig. 3b). These values are markedly lower than those of RuMnCoNi–O (239 mV, 368 mV), Ta–RuO_2_ (258 mV, 384 mV) and C–RuO_2_ (347 mV, 706 mV). The superior performance of RuMnCoNiTa–O is also evident at 50 mA cm^−2^ (Fig. S7), highlighting the synergistic effects of the high-entropy effect and Ta-induced electronic modulation on catalytic activity. Tafel slopes derived from the LSV curves reveal a low Tafel slope of 79.06 mV dec^−1^ for RuMnCoNiTa–O (Fig. S8), which is considerably smaller than those of the other reference catalysts, indicating accelerated reaction kinetics and more favorable charge-transfer behavior. The enhanced intrinsic catalytic activity of RuMnCoNiTa–O is further confirmed by its significantly improved mass activity in Fig. 3c. And at an overpotential of 270 mV (1.5 V *vs.* RHE), RuMnCoNiTa–O delivers a mass activity of 150.84 A g^−1^, substantially outperforming the reference catalysts.^37^

**Fig. 3:**
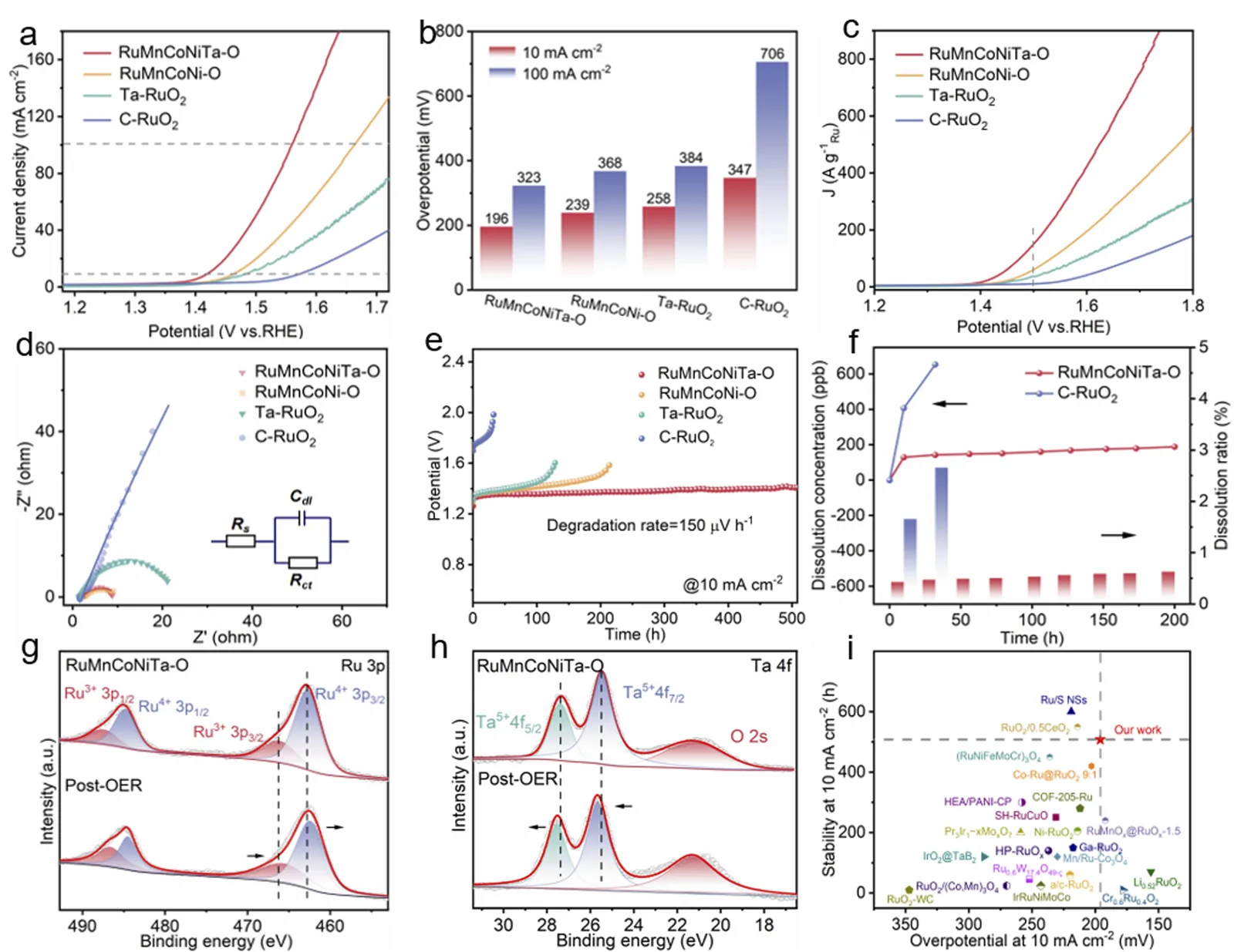
Electrocatalytic oxygen evolution reaction performance in acidic electrolyte (0.5 M H_2_SO_4_). (a) Linear sweep voltammetry (LSV) curves. (b) The corresponding overpotentials of RuMnCoNiTa–O, RuMnCoNi–O, Ta–RuO_2_ and C–RuO_2_ at 10 and 100 mA cm^−2^. (c) Mass activity comparison of the different catalysts. (d) Electrochemical impedance spectroscopy (EIS) Nyquist plots. (e) Long-term chronopotentiometry stability tested at 10 mA cm^−2^. (f) Ru dissolution concentration detected by ICP-OES during the stability test. (g and h) High-resolution XPS spectra of RuMnCoNiTa–O after the OER test for Ru 3p and Ta 4f, respectively. (i) Comparison of the overpotential at 10 mA cm^−2^ with those of reported state-of-the-art Ru-based acidic OER catalysts.

The catalytic kinetics at the electrochemical interface were investigated by electrochemical impedance spectroscopy (EIS) (Fig. 3d). RuMnCoNiTa–O exhibits the smallest charge-transfer resistance (*R*_ct_) among all samples, indicating significantly accelerated electron-transfer kinetics during acidic OER. The electrochemically active surface area (ECSA) was estimated from the double-layer capacitance (*C*_dl_) values derived from cyclic voltammetry measurements conducted at various scan rates within the non-faradaic region in Fig. S9 and S10. The *C*_dl_ of RuMnCoNiTa–O approaches three times that of commercial RuO_2_. Notably, the *C*_dl_ value of RuMnCoNiTa–O (101.89 mF cm^−2^) is much higher than those of C–RuO_2_ (39.03 mF cm^−2^), Ta–RuO_2_ (50.88 mF cm^−2^), and RuMnCoNi–O (61.70 mF cm^−-2^), indicating more catalytically active sites. To isolate intrinsic catalytic kinetics from surface area effects, polarization curves were normalized by *C*_dl_ to evaluate specific activity (*j*_ECSA_). As shown in Fig. S11, ECSA-normalized curves confirm that RuMnCoNiTa–O exhibits 0.50 mA mF^−1^ at 1.5 V *vs.* RHE. This enhancement verifies that its excellent acidic OER performance stems from intrinsic electronic optimization of active sites, rather than a higher active-site density. Local multiaxial lattice strain and optimized orbital hybridization reduce the activation barriers of key LOM intermediates.

The long-term structural stability under harsh acidic conditions is a critical metric for evaluating OER electrocatalysts. And the durability of RuMnCoNiTa–O, RuMnCoNi–O, Ta–RuO_2_, and C–RuO_2_ was evaluated by chronopotentiometry (CP) measurements at a constant current density of 10 mA cm^−2^. As shown in Fig. 3e and Table S2, RuMnCoNiTa–O shows outstanding long-term stability in 0.5 M H_2_SO_4_. Over 500 h of continuous anodic polarization, the catalyst only undergoes mild potential growth. This subtle drift arises from temporary mass-transport limitations arising when evolving oxygen bubbles partially cover active sites, together with mild early-cycle surface restructuring that stabilizes the LOM pathway, rather than irreversible catalyst degradation. Moreover, RuMnCoNiTa–O maintains stable operation for approximately 160 h even at high current densities (100 mA cm^−2^, Fig. S12), further highlighting its outstanding robustness under harsh acidic OER conditions. Such outstanding activity and stability (Fig. S13) originate from the synergistic regulation of the local coordination environment and electronic structure induced by high-entropy coupling and Ta incorporation.

Moreover, after 2000 cyclic voltammetry (CV) cycles, the overpotential of RuMnCoNiTa–O increases by only 7 mV (Fig. S14), demonstrating markedly improved electrochemical durability compared to RuMnCoNi–O without Ta doping. As illustrated in Fig. S15 and S16, the SEM and TEM images of RuMnCoNiTa–O after long-term OER operation reveal that the nanoparticle morphology remains well preserved without noticeable aggregation or structural collapse. Moreover, XRD patterns (Fig. S17) retain the characteristic diffraction peaks of the rutile structure without detectable phase transformation. Inductively coupled plasma optical emission spectrometry (ICP-OES) was used to quantitatively analyze Ru dissolution during the OER process (Fig. 3f). The dissolved Ru concentration of C–RuO_2_ rises rapidly to 407 ppb within 10 h, corresponding to a Ru leaching ratio of 1.66%. In contrast, after 200 h of stability testing, the dissolved Ru concentration of RuMnCoNiTa–O is only 189 ppb, equivalent to only 0.63% mass loss of Ru. The Ru dissolution rate of RuMnCoNiTa–O decreases continuously over the testing period, confirming the high structural integrity of the lattice during catalysis. The stability number (*S*-number) of RuMnCoNiTa–O is enhanced by one order of magnitude compared with C–RuO_2_ and surpasses most reported Ru and Ir-based electrocatalysts (Fig. S18 and Table S3). Such exceptional structural stability can be attributed to the entropy-stabilized lattice distortion effect characteristic of the high-entropy structure.^38,39^ The severe local lattice distortion generates a sluggish diffusion effect, which kinetically suppresses cation migration and increases the energy barrier for metal dissolution from the lattice, thereby effectively stabilizing the Ru–O bond during acidic OER operation.

Post-OER XPS characterization was further conducted to investigate the evolution of the surface electronic structure of RuMnCoNiTa–O after durability testing. High-resolution Ru 3p (Fig. 3g), Mn 2p, Co 2p, and Ni 2p (Fig. S19) spectra show that the characteristic spectral features remain unchanged, while the Ru binding energy exhibits only a slight positive shift, indicating a moderate increase in the Ru oxidation state without obvious surface reconstruction or over-oxidation. Interestingly, the Ta 4f spectra display an opposite trend, exhibiting a slight shift toward lower binding energy after cycling (Fig. 3h). This behavior originates from dynamic electronic redistribution during the electrochemical oxidation process, where the strong electron-withdrawing capability of Ta induces interfacial charge transfer and stabilizes neighboring Ru active centers by regulating the local electron density. Such electronic compensation effectively suppresses excessive oxidation of Ru active sites and mitigates Ru dissolution under harsh acidic conditions. Benefiting from the entropy-stabilized lattice distortion and Ta-induced electronic regulation, the active Ru–O local structure is effectively preserved during prolonged acidic OER operation, ultimately contributing to the exceptional long-term catalytic durability of RuMnCoNiTa–O. Notably, both the peak positions and relative intensities of these three species remain largely unchanged after durability testing in the O 1s spectra. Concurrently, the adsorbed oxygen species (O_ads_), which act as key reaction intermediates, are also retained without significant depletion or phase transformation.

Collectively, these results demonstrate that the synergistic interplay between entropy-stabilized lattice distortion and Ta-induced electronic modulation simultaneously stabilizes both the bulk structure and the surface reactive environment of RuMnCoNiTa–O, thereby intrinsically suppressing Ru leaching and overoxidation.^40^ Overall, RuMnCoNiTa–O exhibits superior OER activity and stability relative to the reference catalysts and most recently reported catalysts (Fig. 3i and Table S4).

### 3.4 Insights into the catalytic reaction mechanism

To elucidate the high-entropy effect, particularly the role of Ta-induced electronic regulation during acidic OER, the reaction mechanism was systematically investigated. Since the lattice oxygen mechanism (LOM) generally exhibits pronounced pH-dependent behavior due to the involvement of decoupled proton–electron transfer steps,^41^ pH-dependent OER measurements were first conducted for all catalysts. As shown in Fig. 4a, RuMnCoNi–O displays a relatively low *ρ*_RHE_ value of 0.58, suggesting limited lattice oxygen participation during the OER process. In contrast, RuMnCoNiTa–O exhibits a stronger pH dependence with an increased *ρ*_RHE_ value of 0.71, indicative of enhanced proton–electron decoupling behavior and a greater contribution from a lattice oxygen-involved pathway.^42^ These results strongly suggest that Ta incorporation promotes lattice oxygen activation during acidic OER. To directly identify reaction intermediates and verify the occurrence of the LOM pathway, *in situ* attenuated total reflection Fourier-transform infrared spectroscopy (*in situ* ATR-FTIR) was performed on both catalysts. For RuMnCoNi–O, spectra collected at increasing potentials only display characteristic absorption bands associated with conventional adsorbate evolution mechanism (AEM) intermediates, including surface-adsorbed *OH (∼3200–3600 cm^−1^) and *OOH (∼1100–1300 cm^−1^), with no detectable signals related to lattice oxygen involvement (Fig. S20). In sharp contrast, RuMnCoNiTa–O exhibits an O–O stretching vibration peak at ∼1400–1600 cm^−1^ under high potentials, assigned to direct O–O bond formation *via* lattice oxygen coupling, a diagnostic intermediate signature for the LOM pathway (Fig. 4b). The attenuated *OOH signal and emerging O–O features collectively confirm that Ta doping triggers a mechanistic switch from the conventional AEM to the LOM, providing direct spectroscopic evidence for subsequent isotope-tracing measurements. To further validate the dominant contribution of the LOM pathway to the high OER activity of RuMnCoNiTa–O, tetramethylammonium cation (TMA^+^) poisoning experiments were performed. TMA^+^ specifically adsorbs onto surface oxygen vacancies, the key active sites for the LOM, thereby blocking the LOM pathway without exerting significant effects on the AEM. As shown in Fig. 4c, the addition of TMA^+^ to the electrolyte induces a marked anodic shift in the OER polarization curve of RuMnCoNiTa–O, with an overpotential increase of 26 mV at 10 mA cm^−2^. The pronounced activity decay upon TMA^+^ adsorption directly confirms that the LOM pathway serves as a critical contributor to the superior OER activity of this catalyst.^43^

**Fig. 4:**
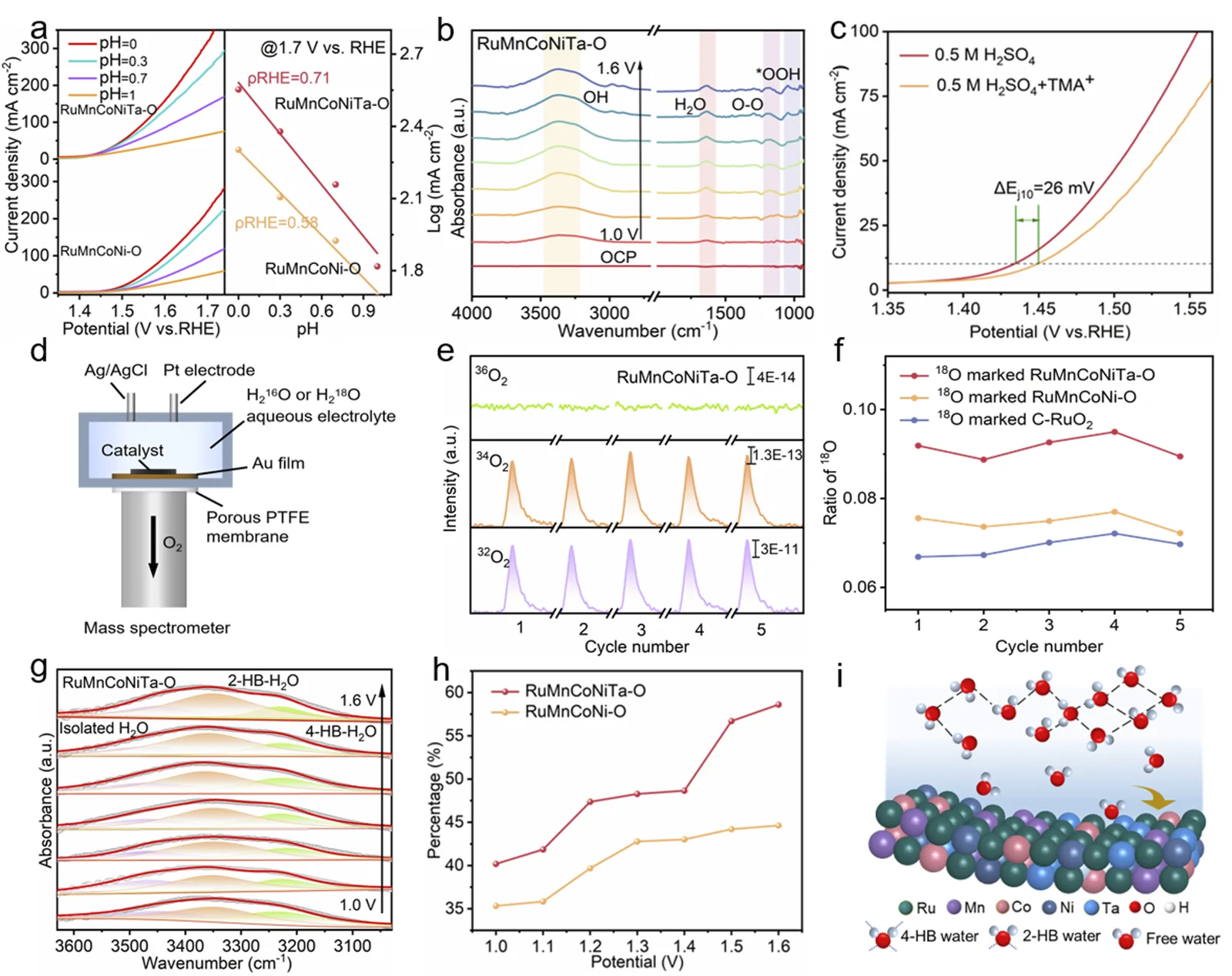
Mechanistic investigations of the OER pathway. (a) pH-dependent OER activity of RuMnCoNiTa–O. (b) *In situ* ATR-SEIRAS of RuMnCoNiTa–O at different operated potentials. (c) OER activity variation before and after TMA^+^ adsorption of RuMnCoNiTa–O. (d) Schematic illustration of ^18^O isotope-labeled differential electrochemical mass spectrometry (DEMS). (e) Online DEMS signals of O_2_ products for RuMnCoNiTa–O. (f) The ratio of ^16^O/^18^O in the gas products of RuMnCoNiTa–O, RuMnCoNi–O and C–RuO_2_. (g) ATR-SEIRAS curves and deconvolution of O–H stretching peaks for RuMnCoNiTa–O. (h) Relative proportions of 2-HB-H_2_O for RuMnCoNiTa–O and RuMnCoNi–O. (i) Schematic illustration of the electrical double layer structure at the interface between the RuMnCoNiTa–O catalyst and the electrolyte.

Differential electrochemical mass spectrometry (DEMS) measurements can provide the most direct evidence for lattice oxygen participation during the OER process. To further elucidate the OER mechanisms of RuMnCoNiTa–O and RuMnCoNi–O, isotope-labeled *operando* DEMS experiments were performed in 0.5 M H_2_SO_4_ electrolytes containing H_2_^18^O and H_2_^16^O (Fig. 4d). C–RuO_2_ exhibits dominant ^32^O_2_ signals with negligible ^34^O_2_ intensity and no detectable ^36^O_2_ signal, indicating minimal lattice oxygen participation and a typical AEM pathway. In comparison, RuMnCoNi–O displays a weak yet discernible ^34^O_2_ signal, suggesting partial lattice oxygen activation and limited involvement of the LOM pathway. In sharp contrast, RuMnCoNiTa–O exhibits significantly enhanced ^34^O_2_ signals accompanied by detectable ^36^O_2_ evolution (Fig. 4e and S21), directly verifying the participation of lattice oxygen in O_2_ generation. Notably, the isotope-labeled oxygen signals remain stable over five consecutive cycles, effectively excluding interference from residual surface-adsorbed ^18^O species and confirming that the detected ^34^O_2_ and ^36^O_2_ originate from bulk lattice oxygen rather than superficial oxygen exchange processes.^44^ Quantitative analysis of the ^18^O fraction in the evolved oxygen further supports this conclusion (Fig. 4f). RuMnCoNiTa–O exhibits the highest ^18^O contribution (∼0.09–0.10), significantly exceeding those of RuMnCoNi–O (∼0.07–0.08) and commercial RuO_2_ (∼0.06–0.07). These results unequivocally demonstrate that Ta incorporation effectively activates lattice oxygen and substantially enhances its participation during acidic OER. Meanwhile, the strengthened Ru–O interaction induced by Ta-mediated electronic modulation helps maintain structural stability during lattice oxygen activation. In addition, the entropy-stabilized high-entropy framework further promotes catalytic activity through synergistic electronic interactions and local coordination reconstruction, ultimately enabling highly efficient and durable acidic OER performance.

Given that proton transfer and interfacial water configuration play essential roles in lattice oxygen-involved reaction pathways, the hydrogen-bond network at the interface was further investigated by *in situ* ATR-FTIR spectroscopy. As shown in Fig. 4g and S22, the O–H stretching region (∼3000–3600 cm^−1^) can be deconvoluted into three characteristic components centered at ∼3500 cm^−1^ (isolated H_2_O), ∼3450 cm^−1^ (two-coordinate hydrogen-bonded water, 2-HB-H_2_O), and ∼3230 cm^−1^ (four-coordinate hydrogen-bonded water, 4-HB-H_2_O), respectively. Compared with strongly hydrogen-bonded 4-HB-H_2_O species, the weakly coordinated 2-HB-H_2_O possesses a more flexible hydrogen-bond network and higher interfacial water mobility, which are beneficial for accelerating proton transfer and facilitating the conversion of oxygen-containing intermediates during the OER.^45^ Notably, RuMnCoNiTa–O exhibits a substantially higher proportion of 2-HB-H_2_O species than RuMnCoNi–O throughout the applied potential range (Fig. 4h), indicating that Ta incorporation effectively reconstructs the interfacial hydrogen-bond network and enriches free-water-like species near the catalyst surface. Such a dynamically hydrated interfacial microenvironment is favorable for rapid proton exchange and lattice oxygen-associated reaction steps, thereby further promoting the LOM-involved OER pathway. The above findings collectively demonstrate that Ta incorporation not only activates lattice oxygen through electronic structure modulation but also optimizes the interfacial proton-transfer environment *via* hydrogen-bond network reconstruction, ultimately contributing to the superior acidic OER performance of RuMnCoNiTa–O (Fig. 4i).

### 3.5 DFT calculation and PEMWE performance

The LOM has attracted extensive attention in acidic OER because it can bypass the conventional AEM pathway by directly involving lattice oxygen in O–O bond formation (Fig. 5a). Such a mechanism circumvents the intrinsic linear scaling relationship between *O and *OOH intermediates, thereby lowering the reaction energy barrier and enhancing catalytic activity. However, continuous lattice oxygen participation is generally accompanied by the dynamic generation and replenishment of oxygen vacancies, which often induces lattice instability, metal dissolution, and catalyst deactivation. To elucidate the fundamental mechanism by which RuMnCoNiTa–O effectively activates the LOM pathway while maintaining excellent structural stability, density functional theory (DFT) calculations were further performed.

**Fig. 5:**
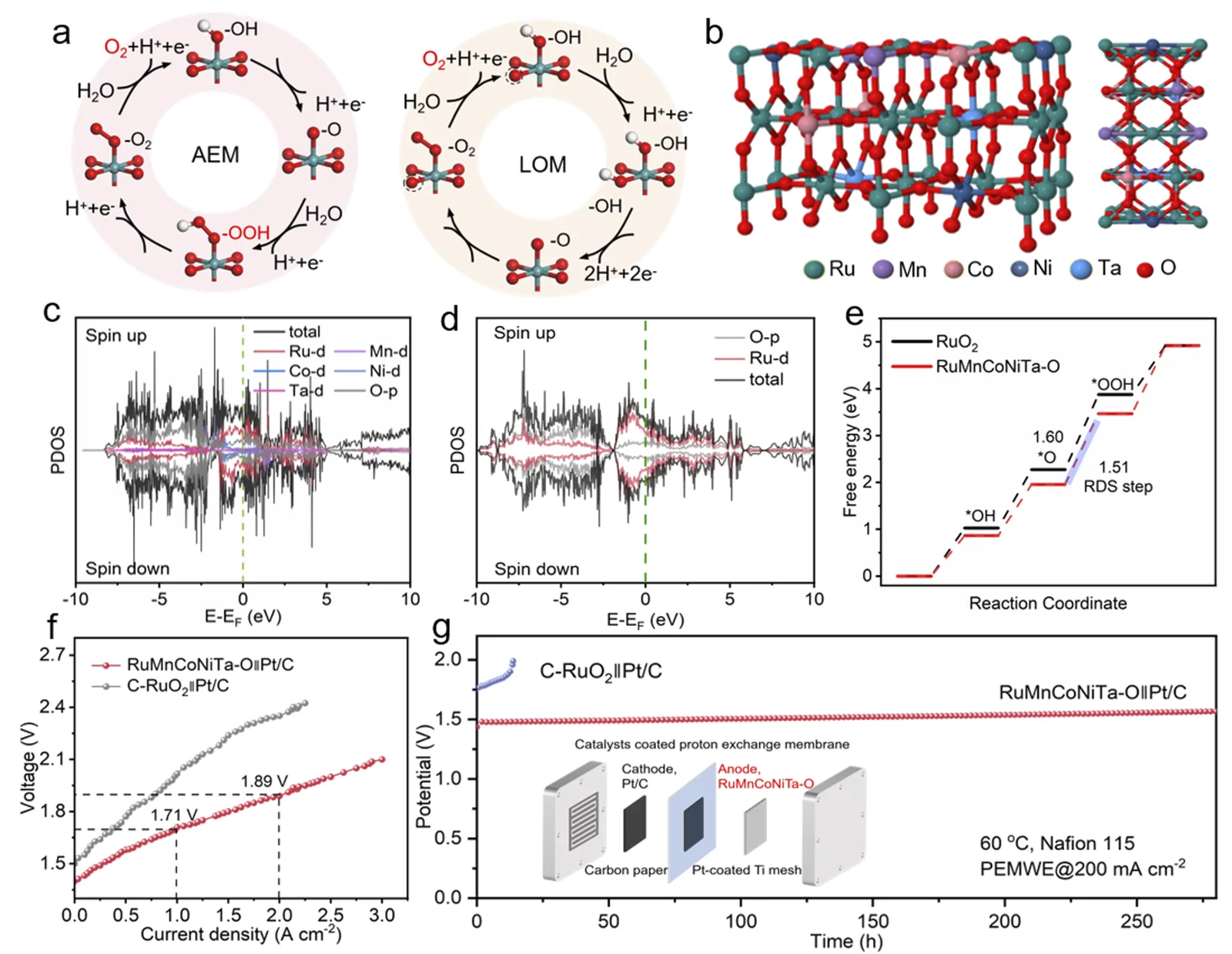
Theoretical insights into the OER mechanism by density functional theory (DFT) calculations. (a) Adsorbate evolution mechanism (AEM) and lattice oxygen-mediated mechanism (LOM). (b) Optimized atomic structure models of RuMnCoNiTa–O. (c and d) Projected density of states (PDOS) for Ru d-orbitals and O p-orbitals. (e) Gibbs free energy diagrams of OER steps under the AEM mechanism. (f) Polarization curves of proton exchange membrane water electrolyzers (PEMWEs). (g) Long-term durability of PEMWEs tested at 60 °C and 200 mA cm^−2^.

Based on the experimental structural characterization through XRD, HRTEM, and ICP analyses, a high-entropy oxide model derived from rutile RuO_2_ was constructed with all five metal species (Ru, Mn, Co, Ni, and Ta) exposed on the catalyst surface. Twelve randomly configured structures were screened according to their total formation energies (Fig. S23), and the thermodynamically most stable configuration was selected for subsequent theoretical investigations. The optimized atomic structures of RuMnCoNiTa–O, RuMnCoNi–O and C–RuO_2_ are shown in Fig. 5b and S24, respectively.

*In situ* Raman spectroscopy was performed across the potential cycle (Fig. S25) to examine structural reversibility and dynamic oxygen vacancy behavior under reaction conditions. For RuMnCoNiTa–O, the characteristic rutile E_g_ and A_1g_ modes attenuate at 1.8 V_RHE_ owing to symmetry breaking from oxygen vacancy formation, yet fully recover their original intensity and profile upon returning to OCP.^46–48^ The RuMnCoNi–O control, however, shows only incomplete spectral recovery, indicating irreversible structural disorder. This *operando* comparison confirms that Ta incorporation stabilizes the rutile framework, enabling reversible oxygen vacancy replenishment during catalytic cycling. To uncover the electronic origin of this structural resilience and lattice oxygen activation, projected density of states (PDOS) analyses were performed for RuMnCoNiTa–O, RuO_2_ and RuMnCoNi–O (Fig. 5c, d and S26). In pure RuO_2_, the overlap between Ru 4d and O 2p orbitals near the Fermi level is relatively limited. In contrast, RuMnCoNiTa–O exhibits significantly enhanced orbital hybridization between Ru 4d and O 2p states around the Fermi level, indicating strengthened Ru–O covalency and more delocalized Ru–O electronic states. To clarify the electronic modulation induced by Ta incorporation, we compared the PDOS of RuMnCoNi–O with RuMnCoNiTa–O. RuMnCoNi–O features Ru 4d states broadly distributed across the Fermi level, with a d-band center of −1.127 eV. High-valence Ta^5+^ doping exerts a strong inductive effect that downshifts the Ru 4d d-band center to −1.085 eV. This modulation strengthens near-Fermi-level Ru 4d and O 2p orbital hybridization, optimizes intermediate binding at active sites, and stabilizes the LOM mechanism against oxidative amorphization and metal dissolution under anodic conditions. Furthermore, to identify the structural origin of Ru–O bond contraction and confirm that this effect arises from Ta incorporation rather than generic high-entropy effects, we used RuMnCoNi–O as a control sample (Fig. S27). Upon introducing high-valence Ta, distinct multiaxial contraction of the Ru–O bonds occurs. This direct computational comparison confirms that Ta acts as a structural anchor that compresses the Ru coordination shell. The shortened interatomic distances strengthen Ru 4d and O 2p hybridization and metal–oxygen covalency, providing a thermodynamic safeguard against irreversible over-oxidation of active sites and metal dissolution under harsh acidic anodic conditions. In particular, the Ta 5d orbitals strongly hybridize with O 2p orbitals, inducing substantial electronic redistribution within the local coordination environment and increasing the oxidation state of neighboring Ru species. Meanwhile, Mn, Co, and Ni further regulate the local electronic distribution by introducing additional electronic states near the Fermi level. Such synergistic electronic interactions are highly consistent with the XPS and XAFS results and provide an electronic foundation for lattice oxygen activation and the promotion of a LOM-involved pathway. Gibbs free energy profiles for the OER compare the reaction energetics of C–RuO_2_ and RuMnCoNiTa–O (Fig. 5e), while the adsorption configurations of key intermediates are illustrated in Fig. S28 and S29. For both catalysts, the conversion of adsorbed *O to *OOH constitutes the rate-determining step (RDS). Notably, the free energy barrier of this critical step is substantially lower for RuMnCoNiTa–O (1.51 eV) than for RuO_2_ (1.60 eV), indicating that the reconstructed electronic structure effectively optimizes the adsorption energetics of oxygen-containing intermediates. Combined with the experimental observations, these results suggest that the synergistic effects of high-entropy engineering and Ta-induced electronic modulation facilitate water activation and proton-coupled oxygen conversion processes, thereby lowering the energy barrier for key intermediate formation and accelerating the overall acidic OER kinetics. To further verify the active sites and clarify the roles of each cation in the quinary oxide, we computed site-specific *OH adsorption energies over all surface metal sites (Table S5). Guided by the Sabatier principle, clear energetic trends appear: Ru delivers a nearly thermoneutral value of −0.039 eV, acting as the thermodynamically optimal active site for fast OER kinetics. Ni (−0.272 eV) and Ta (−0.268 eV) show strong over-binding that poisons surface sites, whereas Mn (+0.155 eV) and Co (+0.786 eV) exhibit under-binding that is unable to stabilize reaction intermediates. These results confirm that Mn, Co and Ni do not serve as direct catalytic centers, but instead electronically optimize the Ru d-band center and maintain the structural integrity of the single-phase lattice.

To evaluate the industrial application potential, a proton exchange membrane water electrolyzer (PEMWE) was assembled with RuMnCoNiTa–O as the anodic catalyst. Polarization curves show that the cell voltages of RuMnCoNiTa–O are only 1.71 V and 1.89 V at industrial-scale current densities of 1.0 A cm^−2^ and 2.0 A cm^−2^, respectively, demonstrating excellent adaptability to industrial operating conditions (Fig. 5f). The long-term stability of the MEA serves as a critical criterion for practical implementation. Long-term durability tests performed at 60 °C and 200 mA cm^−2^ (Fig. 5g) indicate that the MEA can operate stably for around 280 h with negligible performance degradation. This further confirms the outstanding operational stability of RuMnCoNiTa–O under realistic electrolyzer conditions, highlighting its promising application prospects in large-scale green hydrogen production.

## 4. Conclusions

In summary, a Ta-enabled RuMnCoNiTa–O high-entropy oxide was developed as an efficient and durable acidic OER electrocatalyst. Benefiting from entropy-stabilized local lattice distortion and Ta-induced electronic modulation, the catalyst delivers a low overpotential of 196 mV at 10 mA cm^−2^ together with outstanding stability over 500 h in acidic media. Experimental characterization and DFT calculations reveal that Ta incorporation reconstructs the Ru–O electronic environment, enhances Ru–O covalency, and promotes lattice oxygen participation during the OER, while simultaneously suppressing Ru over-oxidation and dissolution. *Operando* spectroscopy and isotope-labeling measurements further confirm the activation of a LOM-involved pathway in RuMnCoNiTa–O. This work demonstrates that regulating local coordination and electronic structures can effectively decouple lattice oxygen activation from catalyst instability, providing a promising strategy for overcoming the activity–stability trade-off in acidic OER catalysis.

## Author contributions

N. Z. supervised the project, coordinated the research, interpreted the results, and reviewed and edited the manuscript. Y. Q. and Y. Y. conceived the study, designed the methodology, performed the experiments, analyzed the data, and wrote the original manuscript. G. Z., J. L., X. W., Y. D., and L. Z. contributed to sample synthesis, characterization, and catalytic performance measurements. H. S. and G. Z. provided technical guidance, assisted with data interpretation, and revised the manuscript. All authors discussed the results and approved the final version of the manuscript.

## Conflicts of interest

There are no conflicts to declare.

## Supplementary Material

SC-017-D6SC05092A-s001

## Data Availability

The data supporting this article have been included in the article and its supplementary information (SI). Additional data are available from the corresponding author upon reasonable request. Supplementary information is available. See DOI: https://doi.org/10.1039/d6sc05092a.
