# Supplementary material for: Regulating lattice oxygen participation through Ta-enabled high-entropy engineering for durable acidic oxygen evolution
Source: Chem Sci. 2026 Jul 31;17(38):18289–301. doi: 10.1039/d6sc05092a (PMC13459051; doi:10.1039/d6sc05092a)
Supplement: Supplementary file 1 [file SC-017-D6SC05092A-s001.pdf]

Supplementary Information (SI) for Chemical Science.
This journal is © The Royal Society of Chemistry 2026

Supporting Information

Regulating Lattice Oxygen Participation through Ta-Enabled High-Entropy Engineering for Durable Acidic Oxygen Evolution

Yingdi Qiu[1,2,#], Yinze yang[2,#], Gemaming Zhang[1,2], Juan Li[1,2], Xiaochen Wang[2], Yibo Dang[1,2], Lili Zhang[2], Huishan Shang[2,], Guoli Zhou[1,*], Ning Zhang[1,2,*]*

[1] State Key Laboratory of Critical Metals Beneficiation, Metallurgy and Purification, Zhengzhou University, Zhengzhou 450001, P. R. China.

[2] School of Chemical Engineering, Zhengzhou University, Zhengzhou 450001, P. R. China.

[#] These authors contributed to the work equally.

[*]Corresponding Author: Ning Zhang, Huishan Shang, Guoli Zhou.

E-mail: nzhang@zzu.edu.cn, shanghs@zzu.edu.cn, zglcumt@126.com.

1 Experimental section

1.1 Materials

All chemicals were obtained from commercial suppliers at analytical grade and used as received without further purification. $RuCl_3$ (99.9% metals basis), $MnCl_4 \cdot 4H_2O$ (99.0%), Carbon paper, the reference commercial RuO_2 (99.9% metals basis) were purchased from Sigma-Aldrich Co., Ltd. $NiCl_2 \cdot 6H_2O$ (98.0%) was purchased from Tianjin Fengchuan Chemical Reagent Technology Co., Ltd. $CoCl_2 \cdot 6H_2O$, $TaCl_5$ (99.8%) were purchased from Aladdin Chemical Co., Ltd. $NaNO_3$ (≥99.0%) was purchased from Tianjin Komeo Chemical Reagent Co., Ltd. Sulfuric acid (H_2SO_4, 97%) was purchased from Xilong Science Co., Ltd. The 40% Pt/C was purchased from Johnson Matthey Co., Ltd. Nafion 115 and 5 wt% Nafion ionomer was purchased from DuPont Co., Ltd.

1.2 Characterization

X-ray diffraction (XRD) patterns were performed on Bruker D8 Advance. Scanning electron microscopy (SEM) images were carried out on a JSM-7500F field emission scanning electron microscope with operating voltages of 5 kV. The transmission electron microscopy (TEM) images were observed using a FEI Talos F200S emission scanning electron microscope. X-ray photoelectron spectroscopy (XPS) tests were obtained from an Escalab 250Xi electron spectrometer with Al Kα radiation. All spectra were calibrated by using the C 1s (binding energy of 284.8 eV) as a reference point. The ratio of Ru/Mn/Co/Ni/Ta in the as-prepared samples and the concentration of dissolved metal ions after the OER process in the electrolyte were analyzed by inductively coupled plasma optical emission spectroscopy (ICP-OES, Thermo iCAP 9820). In situ ATR-FTIR measurements were conducted on a BRUKER-Fourier Transform Infrared Spectrometer-INVENIOS.

1.3 Electrochemical measurements

All electrochemical measurements were performed on a CHI760E electrochemical workstation (Shanghai Chenhua, China) at room temperature using a standard three-electrode setup system. 1 mg of catalyst and 10 μL of 5 wt% Nafion solution were dispersed in 90 μL of a mixed solvent of water and isopropanol with a volume ratio of 1:3. After 30 min of ultrasonic treatment, the desired ink was dropped onto a carbon paper (electrode surface area: 0.5 cm^2), followed by natural drying, and then used as the working electrode. A platinum sheet electrode was used as the counter electrode, and a saturated Ag/AgCl electrode was used as counter and reference electrodes. Before each electrochemical measurement, the electrocatalyst was

activated, and the linear sweep voltammetry (LSV) curves were recorded at a scan rate of 10 mV s^{-1}. The final potential was calibrated into reversible hydrogen electrode (RHE) using the equation: $E_{RHE} = E_{Ag/AgCl} + 0.0592$ pH + 0.197 (0.5 mol L^{-1} H_2SO_4, pH = 0). Tafel plots were constructed by sweeping the electrode potential from high to low values across the linear Tafel region. Electrochemical impedance spectroscopy (EIS) was acquired over a frequency range of 0.01 - 10^5 Hz. The double-layer capacitance was obtained by scanning in the non-Faradaic region (from -0.1 to 0.1 V *vs.* RHE) at different scan rates (30, 40, 50, 60, and 70 mV·s^{-1}). The durability of RuMnCoNiTa-O and other reference samples was tested at current densities of 10 mA·cm^{-2} and 100 mA·cm^{-2}.

1.4 Electrochemical measurements in PEM electrolyzer

To construct the membrane electrode assembly (MEA), RuMnCoNiTa-O was used as the anode catalyst, and commercial Pt/C (40 wt%) was employed as the cathode catalyst. For the preparation of anode and cathode catalyst inks, the ball-milled catalyst was dispersed in a mixed solution of isopropanol and deionized water with a volume ratio of 3:1, followed by the addition of 5 wt% Nafion solution and ultrasonic treatment for at least 30 min to obtain a homogeneous catalyst ink. The prepared cathode and anode catalyst inks were uniformly sprayed onto the surface of polytetrafluoroethylene (PTFE) films at 80 °C. Subsequently, the PTFE membrane coated with Pt/C, Nafion 115 proton exchange membrane, and the PTFE membrane coated with RuMnCoNiTa-O were hot-pressed under controlled temperature and pressure. After peeling off the surface PTFE membranes, the membrane electrode with catalytic layers was finally fabricated. After optimizing the catalyst loading, the loadings of the anode and cathode catalysts were controlled at 2 mg·cm^{-2} and 1 mg·cm^{-2}, respectively. To construct a PEM electrolyzer for performance evaluation, a titanium felt was adopted as the anode porous transport layer (PTL), and carbon paper was used as the cathode. The assembly torque of the fixture was predefined, and the effective area of the electrode was determined to be 4.84 cm^2. The PEM electrolyzer was operated at 60 °C with deionized water as the reaction solvent, and the flow rate was controlled at 50 mL·min^{-1}. Polarization curves were collected in the current density range of 0 to 3 A·cm^{-2}. The stability of the PEM electrolyzer was evaluated by performing a chronopotentiometry test at a constant current density of 200 mA·cm^{-2} for 280 h.

1.5 Isotope labeling experiments

DEMS experiments were all performed in a sealed two-electrode electrolytic cell system, where a platinum-plated titanium mesh and catalyst-loaded carbon paper were used as the

cathode and anode, respectively. Prior to conducting DEMS measurements, the catalyst was first subjected to ^{18}O isotope labeling treatment: the catalyst was placed in a 0.5 mol L^{-1} sulfuric acid solution labeled with ^{18}O, and a chronopotentiometry test was carried out at an applied current density of 10 mA·cm^{-2} for 10 min.

After the labeling was completed, the catalyst was thoroughly washed with deionized water ($H_2{}^{16}O$) and then transferred to a vacuum oven for constant-temperature drying for 1 h to completely remove the physically adsorbed $H_2{}^{18}O$ on the catalyst surface, thereby avoiding its interference with subsequent test results. After completing the above pretreatment steps, the online DEMS measurement was initiated.

During the measurement, a three-intermittent chronopotentiometry method was adopted, maintaining a constant current density of 10 mA·cm^{-2}. The gaseous reaction products were detected in real time by a mass spectrometer, and characteristic mass spectral signals including ^{32}O, ^{34}O and ^{36}O were successfully captured. To eliminate the experimental error caused by the natural abundance of ^{18}O isotope, C-RuO_2 was used as a reference sample in this study to conduct control experiments.

1.6 Calculation of the specific current density per electrochemically active surface

Electrochemically active surface areas (ECSAs) were determined from the electrochemical double-layer capacitance of the catalytic surface. The double-layer capacitance (C_{dl}) was quantitatively calculated by analyzing the double-layer charging curves recorded via cyclic voltammetry in the non-Faradaic potential region at scan rates of 30 to 70 mV s^{-1}. The C_{dl} value was derived using the following equation：

$$j = v\,Cdl \quad \text{(Equation 1)}$$

j denotes the non-Faradaic capacitive current extracted from cyclic voltammetry (CV) curves, and v represents the scan rate. The double-layer capacitance (C_{dl}) is determined from the slope of the j-v plot. The electrochemically active surface area (ECSA) is calculated from the double-layer capacitance using the following relationship:

$$ECSA = Cdl/Cs \quad \text{(Equation 2)}$$

C_S denotes the specific capacitance of the sample, which has been determined as 0.035 mF cm^{-2} in accordance with previous literature reports.

1.7 Calculation of the mass activity

The mass activity (j_{mass}) of Ru-based catalysts was determined using the following equation:

$$jmass = \frac{igeo}{mcat \times Ru\ wt\%} \quad \text{(Equation 3)}$$

i_{geo} represents the geometric current measured by linear sweep voltammetry (LSV), m_{cat} represents the catalyst weight loaded on the electrode, and Ru wt% refers to the mass fraction of Ru in the catalysts, which is calculated from the results determined by inductively coupled plasma optical emission spectrometry (ICP-OES).

1.8 Calculation of S-number

S-number was calculated by the following equation as previously reported:

$$S\text{-}number = \frac{nO2}{nRudissolved} \quad \text{(Equation 4)}$$

n_{O_2} refers to the total amount of evolved O_2, calculated from the total quantity of charge transferred. $n_{Rudissolved}$ denotes the dissolved Ru content determined by inductively coupled plasma optical emission spectrometry (ICP-OES).

1.9 Theoretical calculation details

Spin-polarized density functional theory (DFT) calculations were performed using the Vienna Ab initio Simulation Package (VASP) code [1]. The exchange-correlation functional was treated within the generalized gradient approximation (GGA) parameterized by Perdew-Burke-Ernzerhof (PBE) [2]. The core-valence interactions were described by the projector augmented-wave (PAW) method. A plane-wave basis set was employed with a kinetic energy cutoff of 500 eV [3]. To accurately capture the non-local long-range interactions, the Grimme's DFT-D3 semi-empirical dispersion correction with Becke-Johnson (BJ) damping was incorporated in all calculations. The electronic self-consistent field (SCF) cycles were converged to a tolerance of 10^{-6} eV. Structural relaxations were conducted using the conjugate gradient algorithm (or RMM-DIIS depending on the ALGO setting) until the Hellmann-Feynman forces on each active atom were less than 0.02 eV/Å [4]. To ensure computational efficiency while maintaining accuracy, the Brillouin zone was sampled with a Gamma-centered Monkhorst-Pack K-point grid, specifically optimized for the slab dimensions.

The (110) surface of rutile RuO_2 and the corresponding Ru-Mn-Co-Ni-Ta high-entropy oxides (HEOs) were modeled using a slab geometry. To prevent spurious interactions between periodic images, a vacuum region of 15 Å was applied along the direction perpendicular to the surface. During optimization, a selective dynamics scheme was utilized: the bottom atomic layers were constrained to their optimized bulk coordinates to represent the underlying bulk

environment, while the uppermost layers and the adsorbed species were fully relaxed to their minimum energy configurations.

The Gibbs free energies of the oxygen evolution reaction (OER) intermediates were calculated based on the computational hydrogen electrode (CHE) model. The free energy change for each elemental step was calculated using the following equation [5]:

$$\Delta G = \Delta E_{DFT} + \Delta ZPE - T\Delta S \quad \text{(Equation 5)}$$

where ΔE_{DFT} is the total energy difference directly obtained from DFT calculations, ΔZPE is the zero-point energy difference, and ΔS is the change in entropy. The ZPE and entropy of the adsorbed species were derived from vibrational frequency calculations, while those for free gas molecules were taken from standard thermodynamic databases at 298.15 K.

To avoid the well-known errors of standard GGA functionals in calculating the O_2 triplet ground state, the free energy of an O_2 molecule (G_{O2}) was derived using the experimental free energy of water splitting ($H_2O \rightarrow 1/2O_2 + H_2$, $\Delta G = 2.46$ eV),

yielding $\Delta O_2 = 4.92 + 2G_{H2O} - 2G_{H2}$ (Equation 6)

The OER performance was investigated *via* the conventional AEM. The reaction proceeds through four consecutive proton-coupled electron transfer (PCET) steps on a single metal active site (M):

$$* + H_2O(l) \rightarrow *OH + H^+ + e^- \quad \text{(Equation 7)}$$

$$*OH \rightarrow *O + H^+ + e^- \quad \text{(Equation 8)}$$

$$*O + H_2O(l) \rightarrow *OOH + H^+ + e^- \quad \text{(Equation 9)}$$

$$*OOH \rightarrow * + O_2(g) + H^+ + e^- \quad \text{(Equation 10)}$$

The theoretical overpotential (η) was derived from the free energy change of the potential-determining step (PDS), defined as:

$$\eta = \max \frac{\{\Delta G1,\ \Delta G2,\ \Delta G3,\ \Delta G4\}}{e} - 1.23\ eV \quad \text{(Equation 11)}$$

2 Supporting Figures

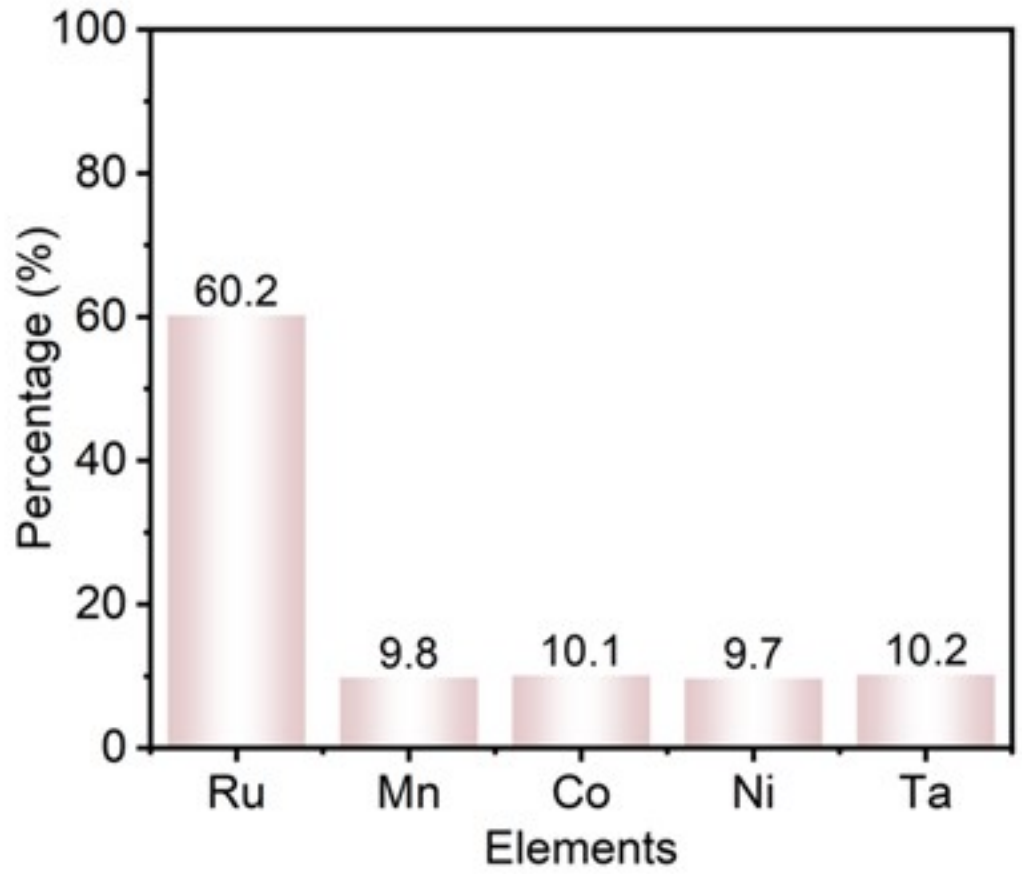

Figure S1. The atomic ratios of metal components in RuMnCoNiTa-O.

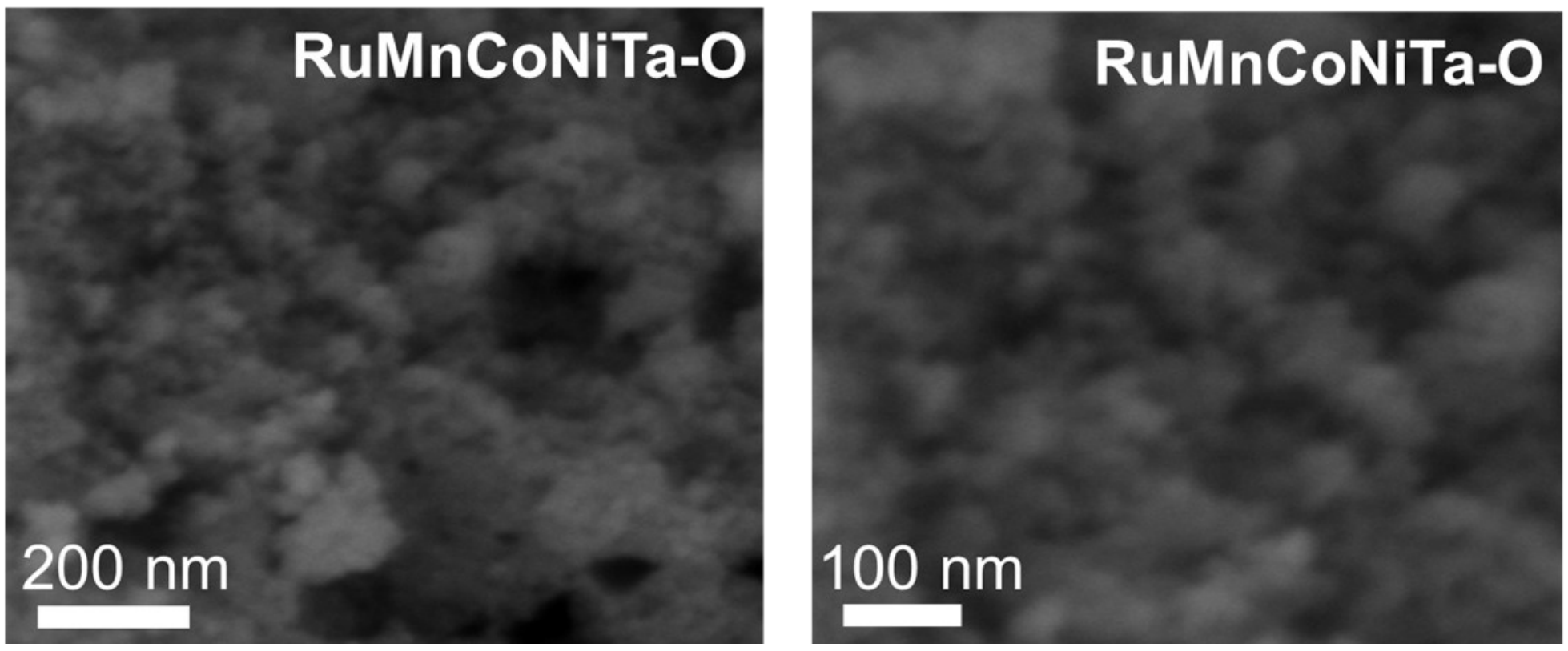

Figure S2. SEM images of RuMnCoNiTa-O.

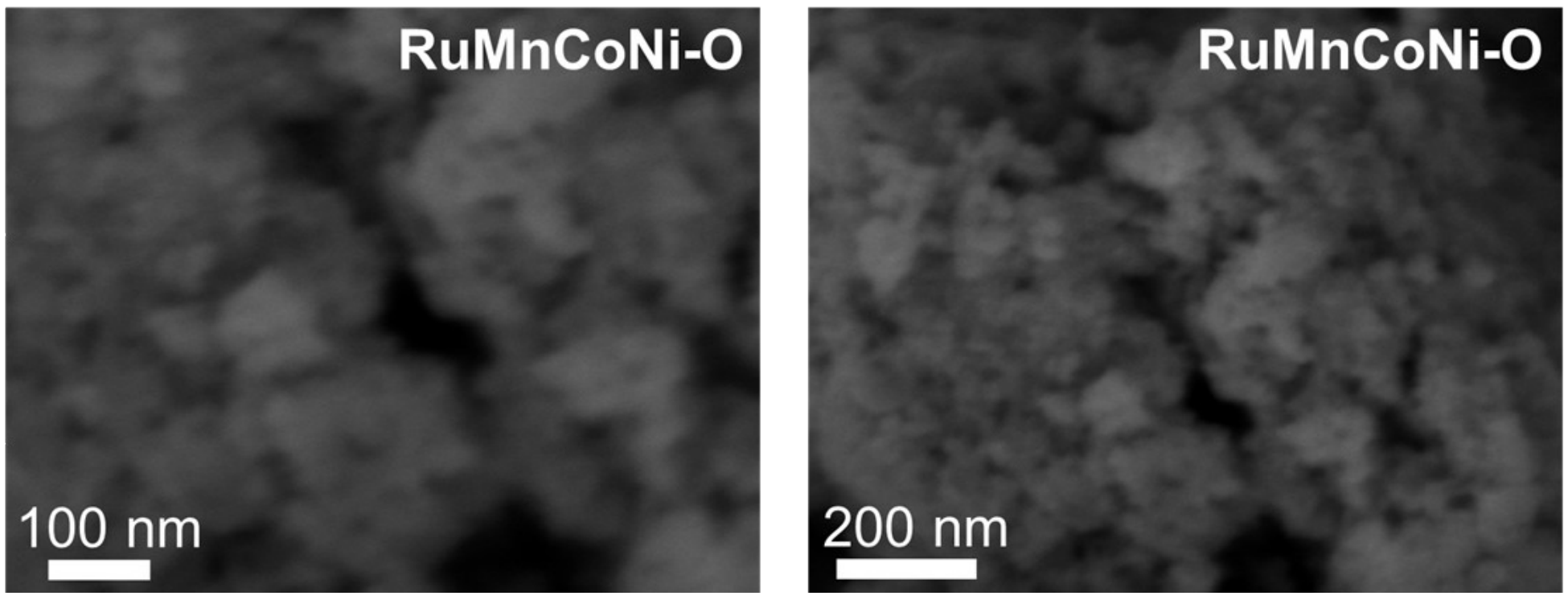

Figure S3. SEM images of RuMnCoNi-O.

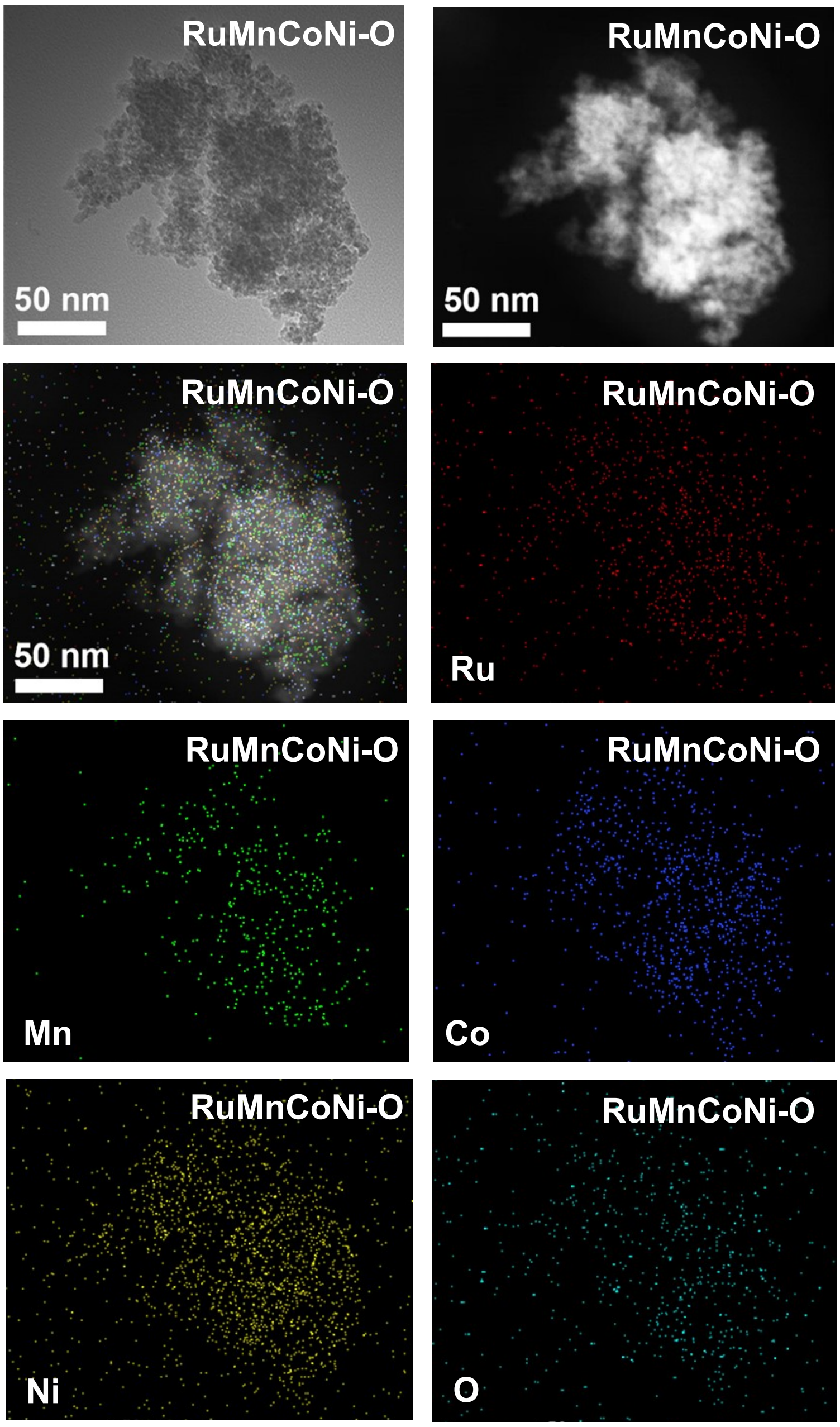

Figure S4. TEM images of RuMnCoNi-O and EDS of Ru, Mn, Co, Ni and O.

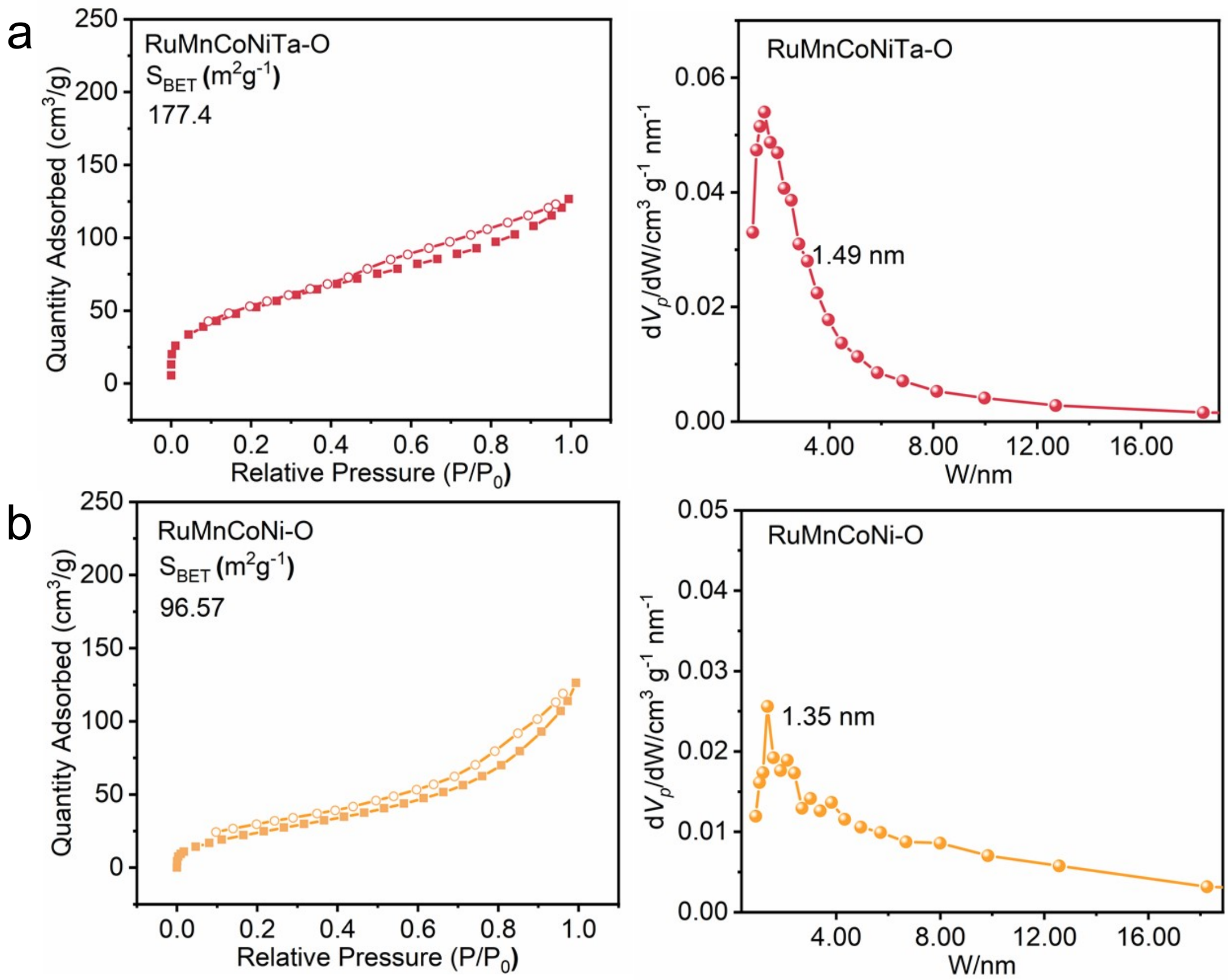

Figure S5. N_2 isothermal adsorption curves and pore size distribution of (a) RuMnCoNiTa-O and (b) RuMnCoNi-O.

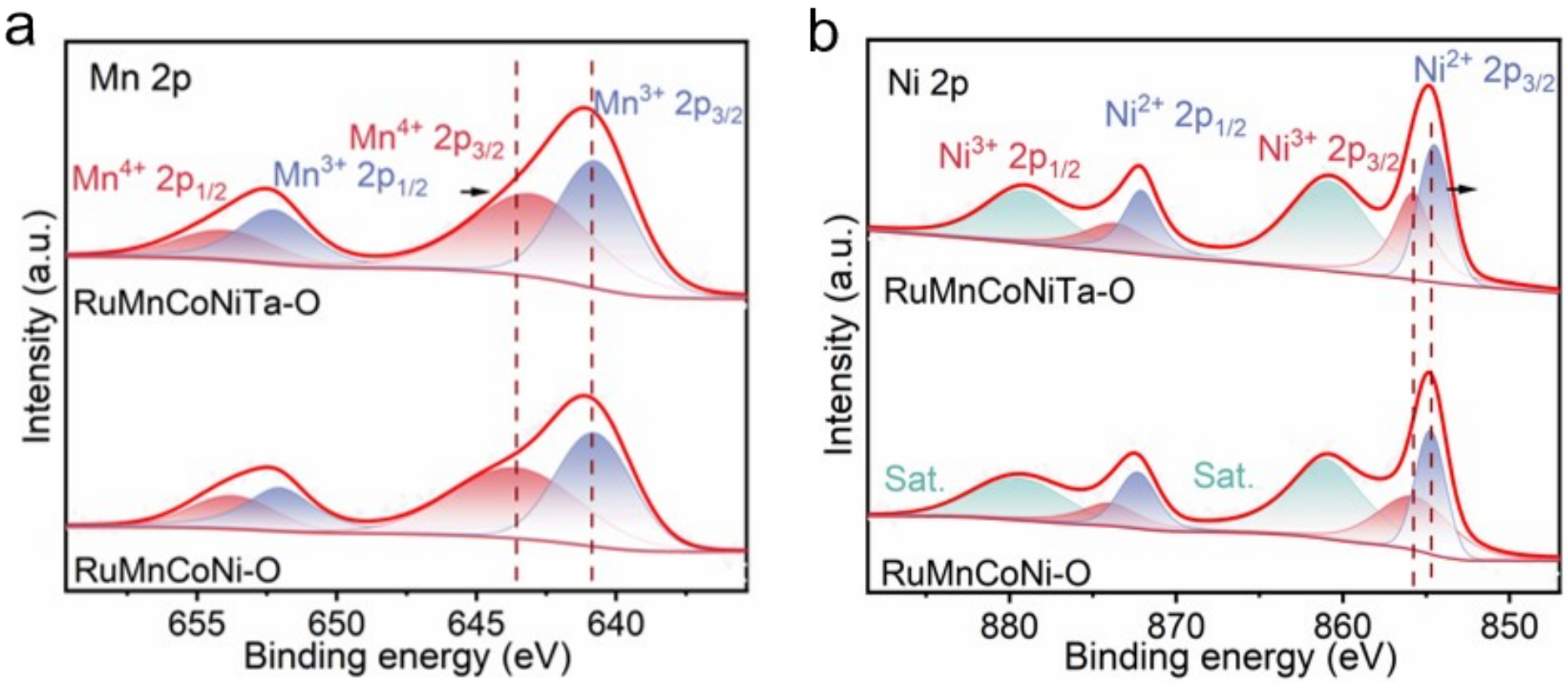

Figure S6. Comparison of Mn 2p and Ni 2p XPS spectra of RuMnCoNiTa-O and RuMnCoNi-O.

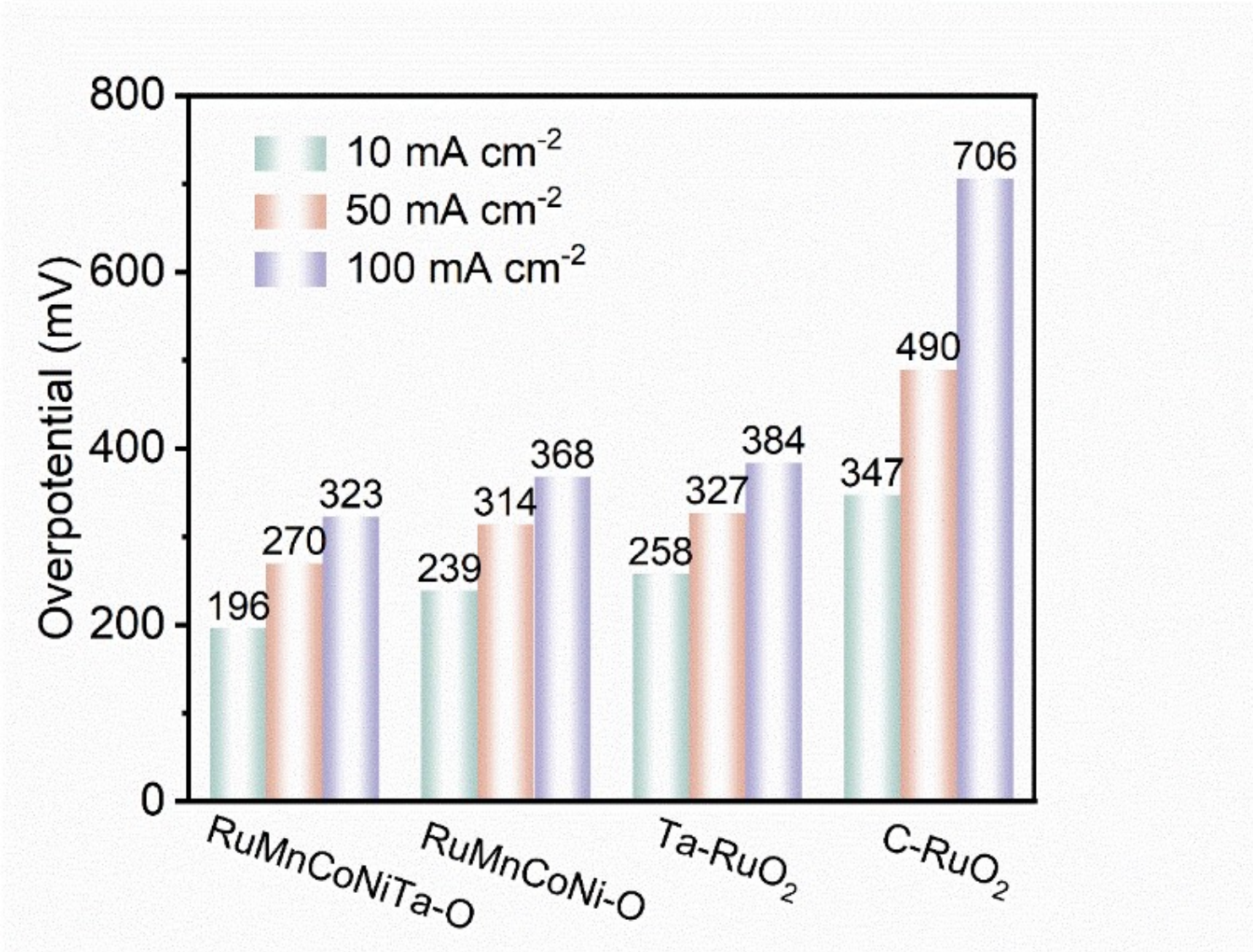

Figure S7. The corresponding overpotential of RuMnCoNiTa-O, RuMnCoNi-O, Ta-RuO_2 and C-RuO_2 at 10, 50 and 100 mA cm^{-2}.

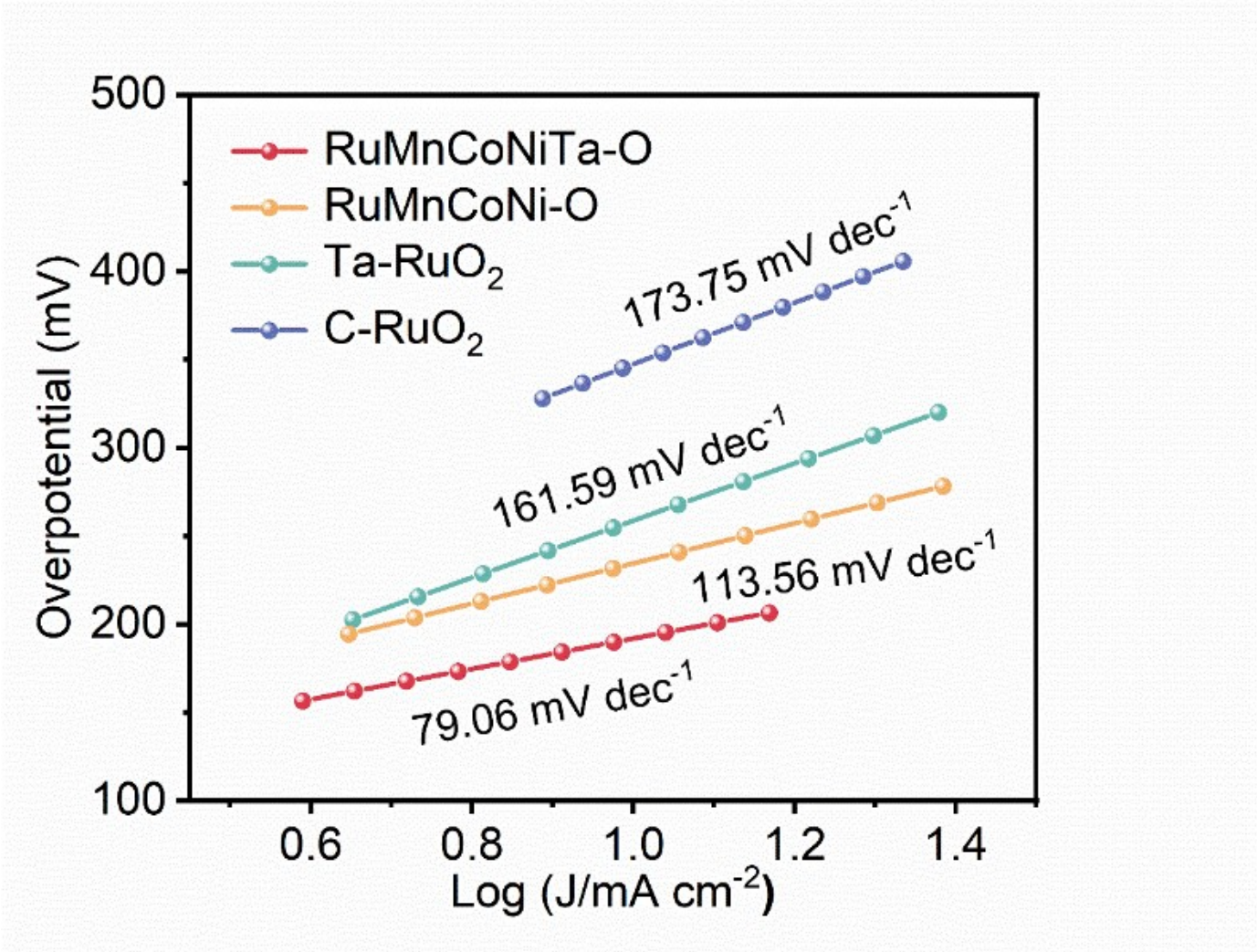

Figure S8. Corresponding Tafel slopes derived from LSV for the materials.

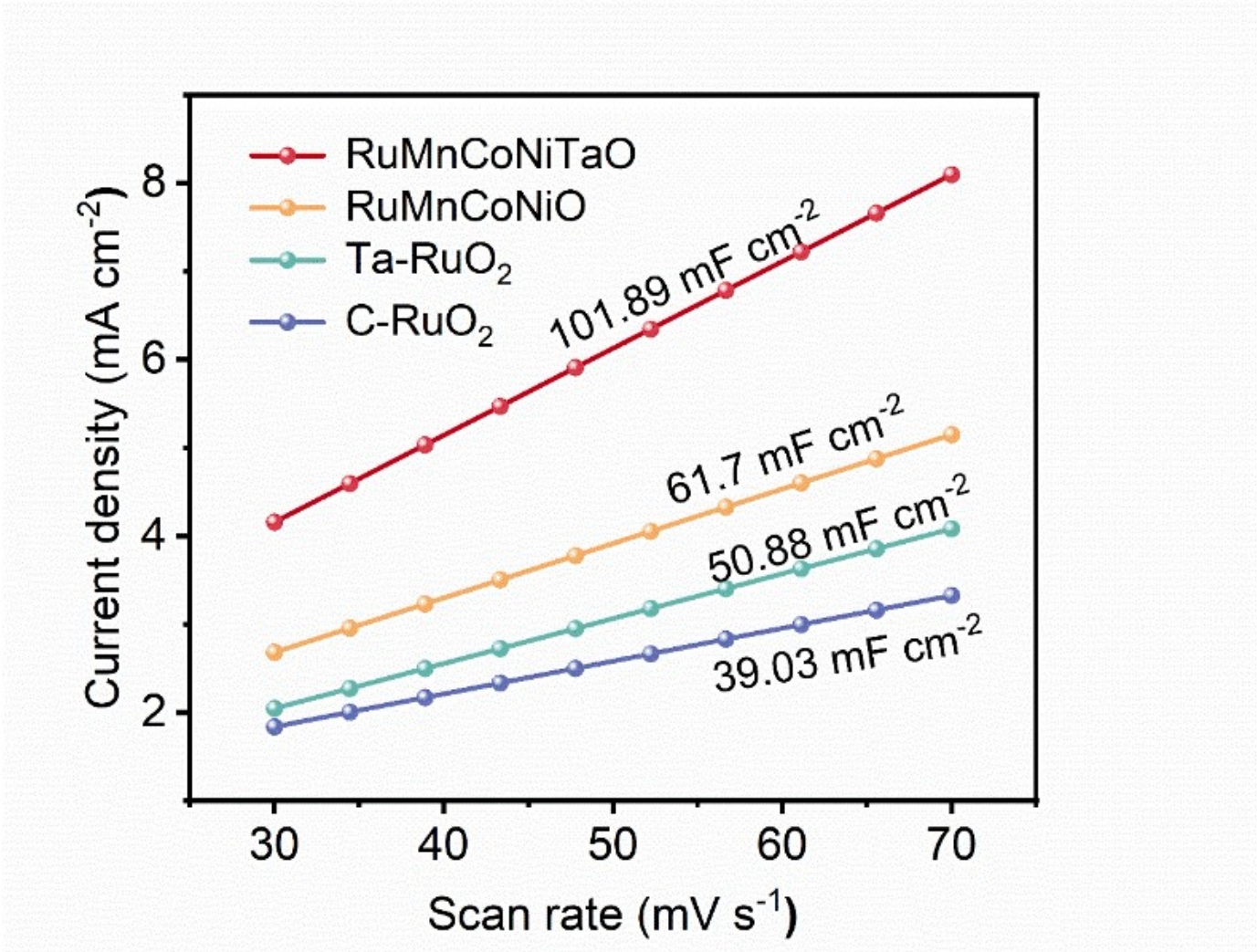

Figure S9. C_{dl} values of various electrocatalysts extracted from CV curves at different scan rates.

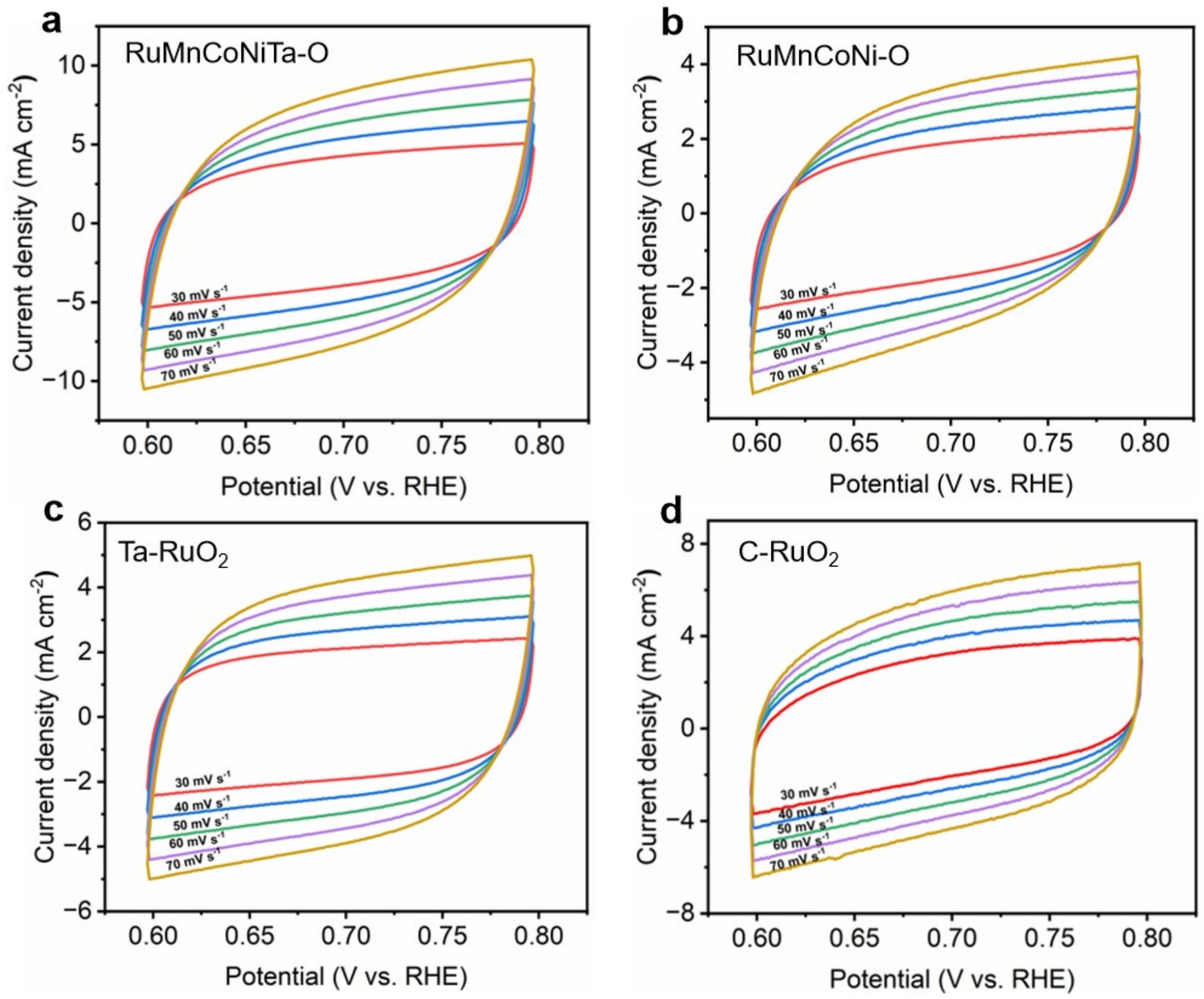

Figure S10. CV curves at different scan rates of RuMnCoNiTa-O, RuMnCoNi-O, Ta-RuO$_2$ and C-RuO$_2$ in 0.5 M H_2SO_4 solution.

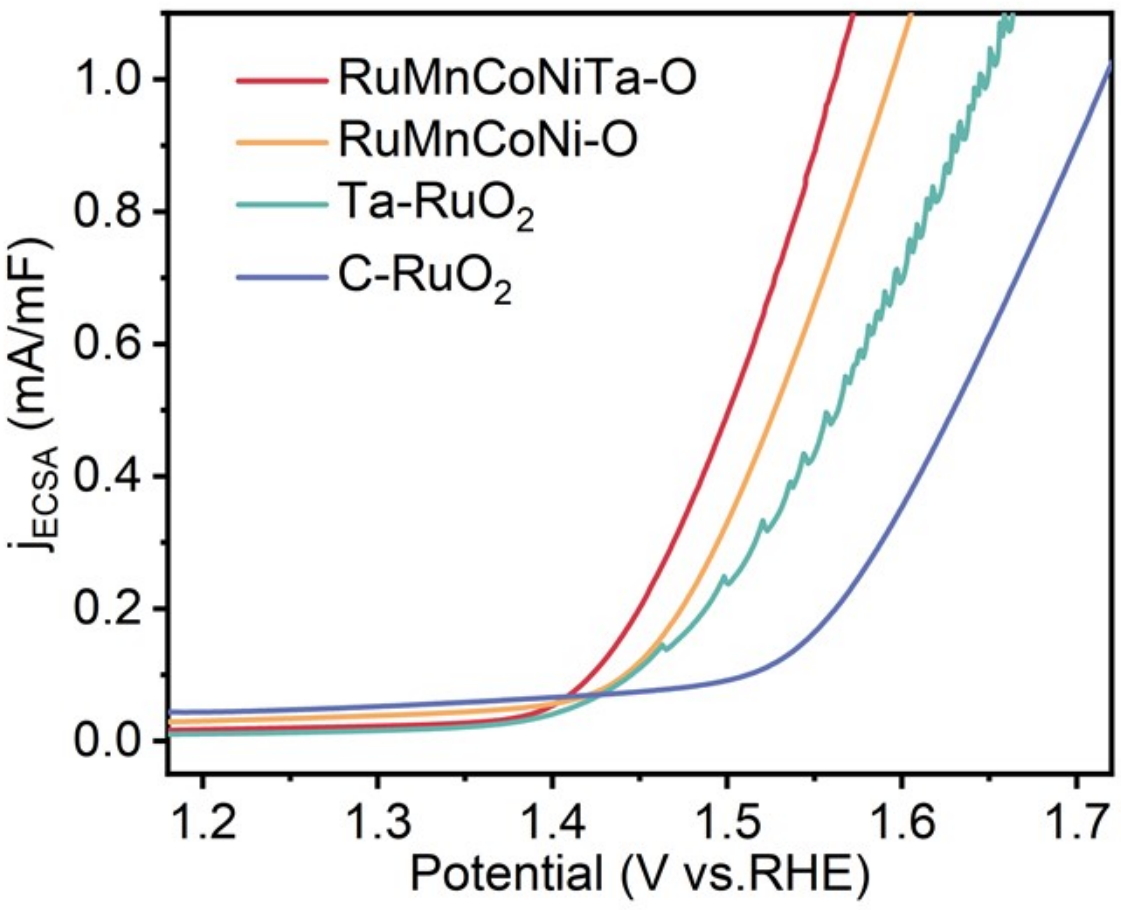

Figure S11. Electrochemical active surface area (ECSA)-normalized LSV curves.

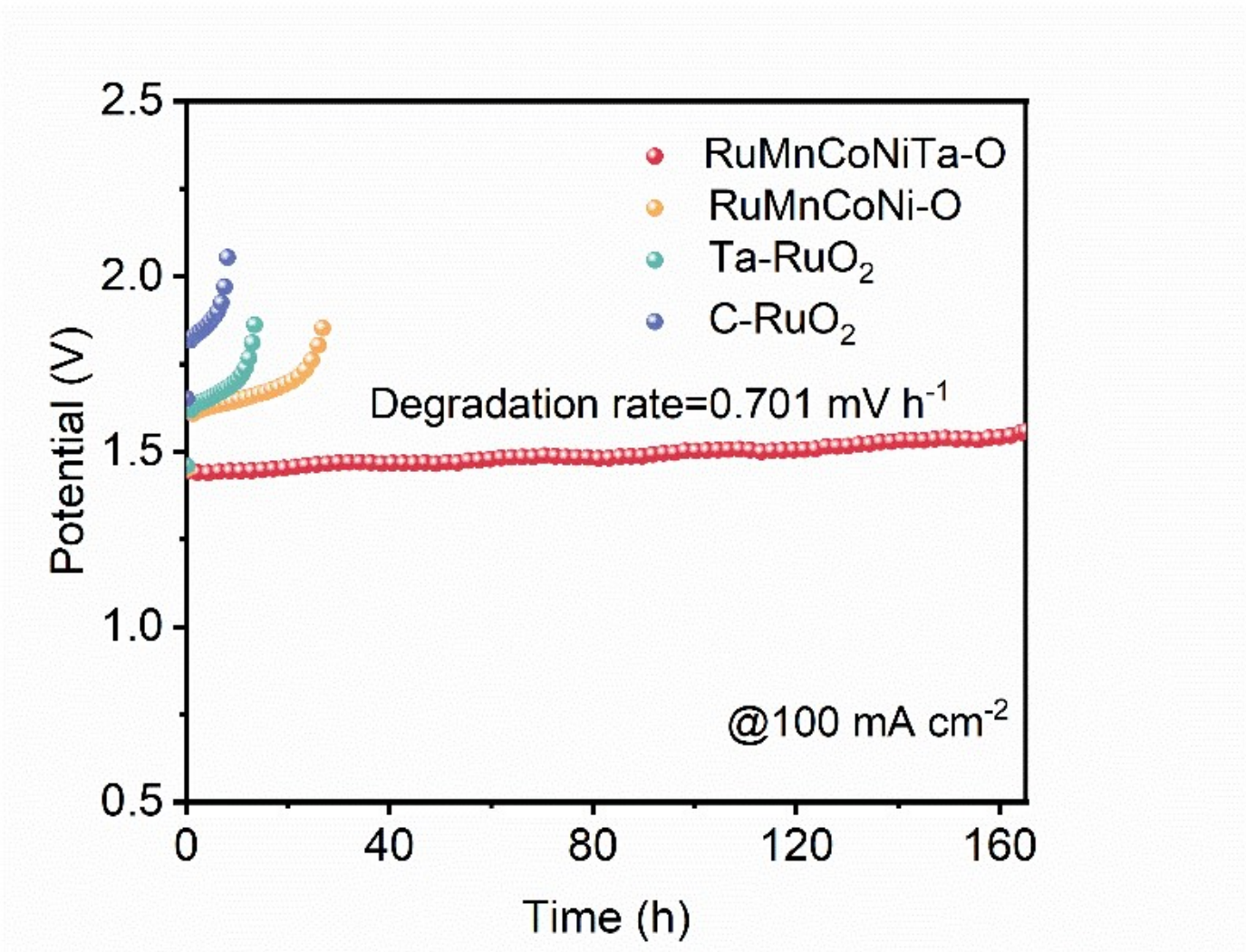

Figure S12. Long-term chronopotentiometry stability of the materials tested at 100 mA cm^{-2}.

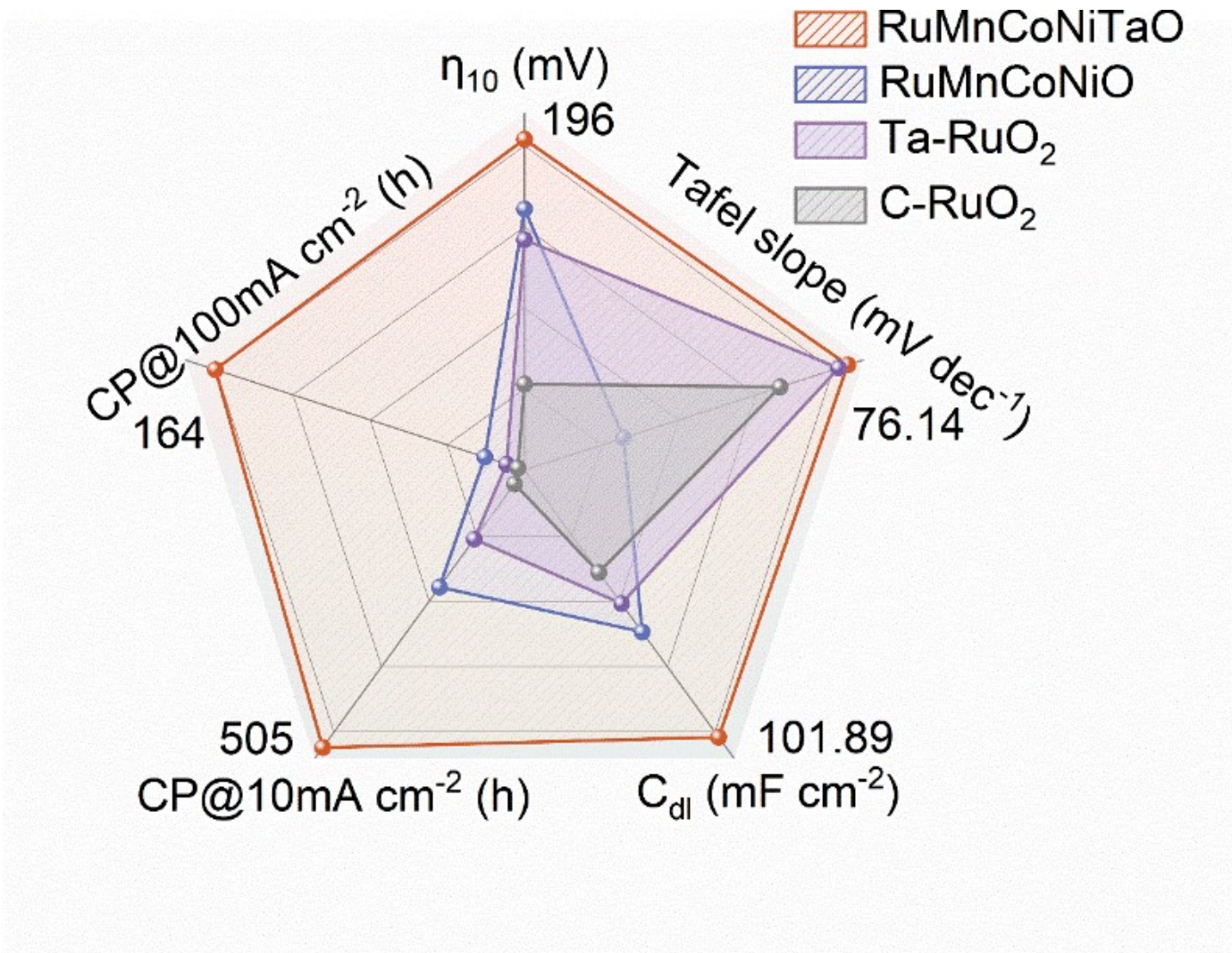

Figure S13. Comparison of overpotential at 10 mA cm^{-2}, Tafel slopes, C_{dl}, CP@10 and 100 mA cm^{-2}.

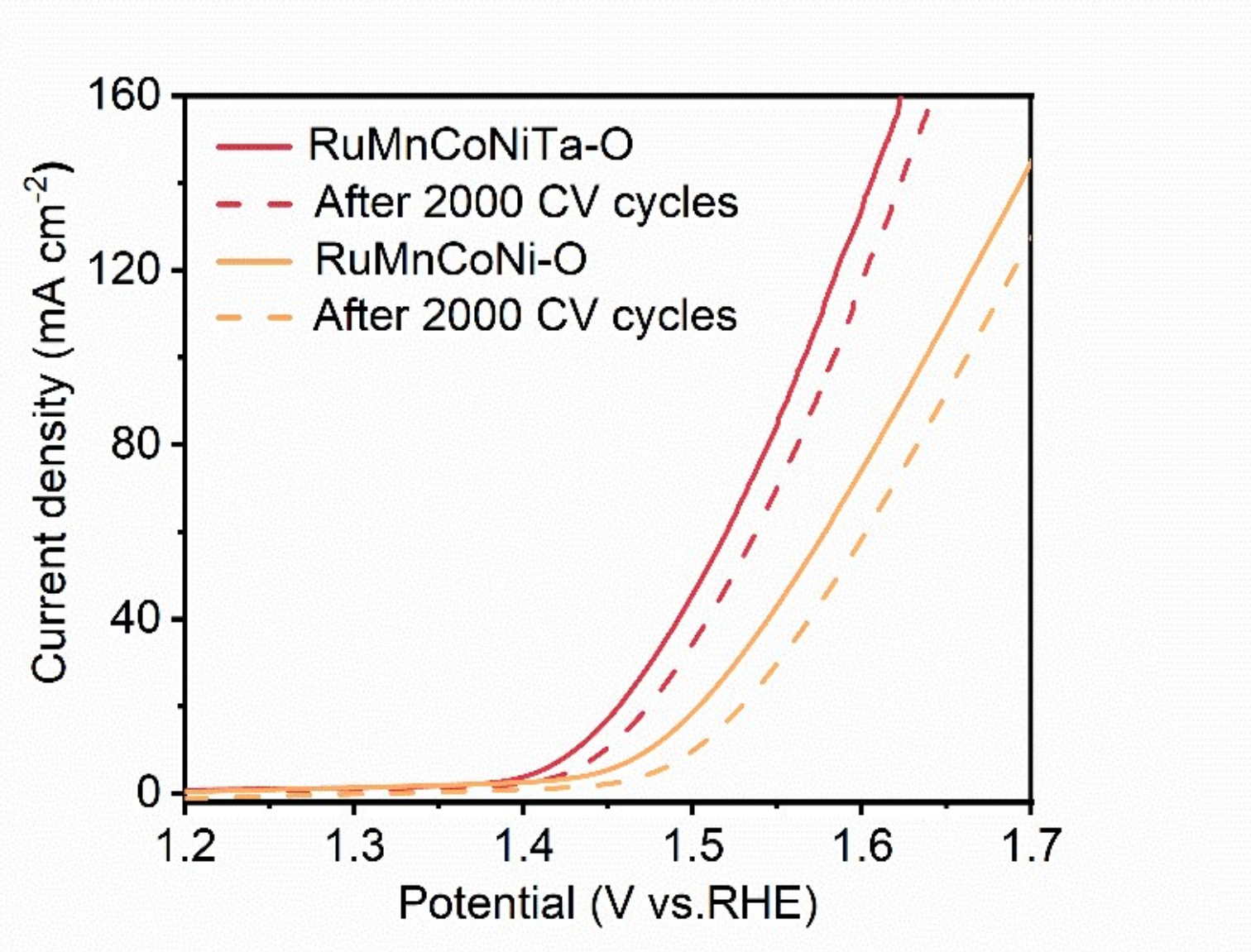

Figure S14. LSV curves of RuMnCoNiTa-O and RuMnCoNi-O before and after 2000 CV cycles test.

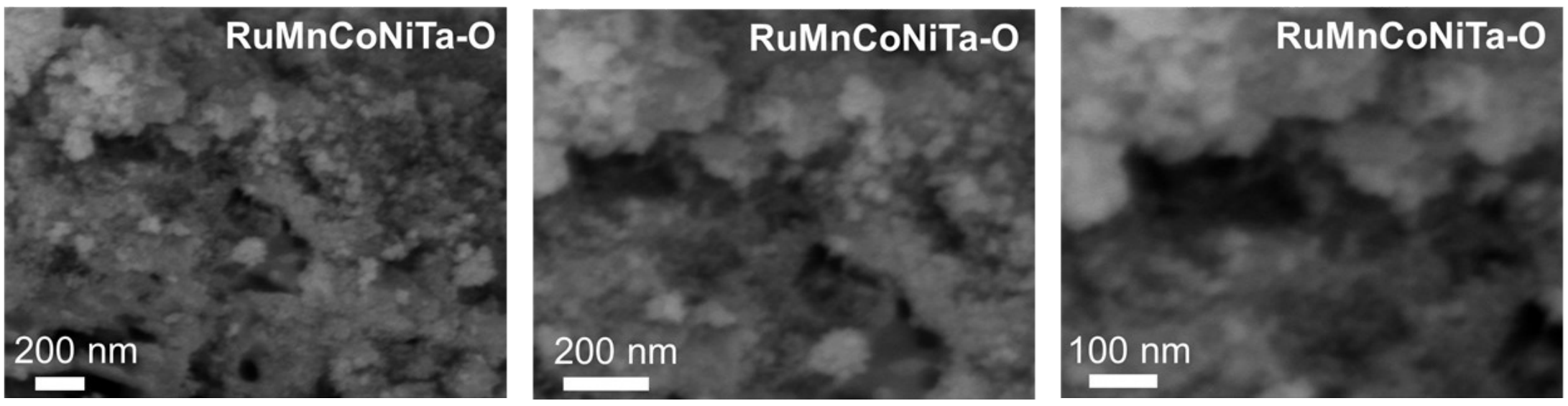

Figure S15. SEM images of RuMnCoNiTa-O after OER test.

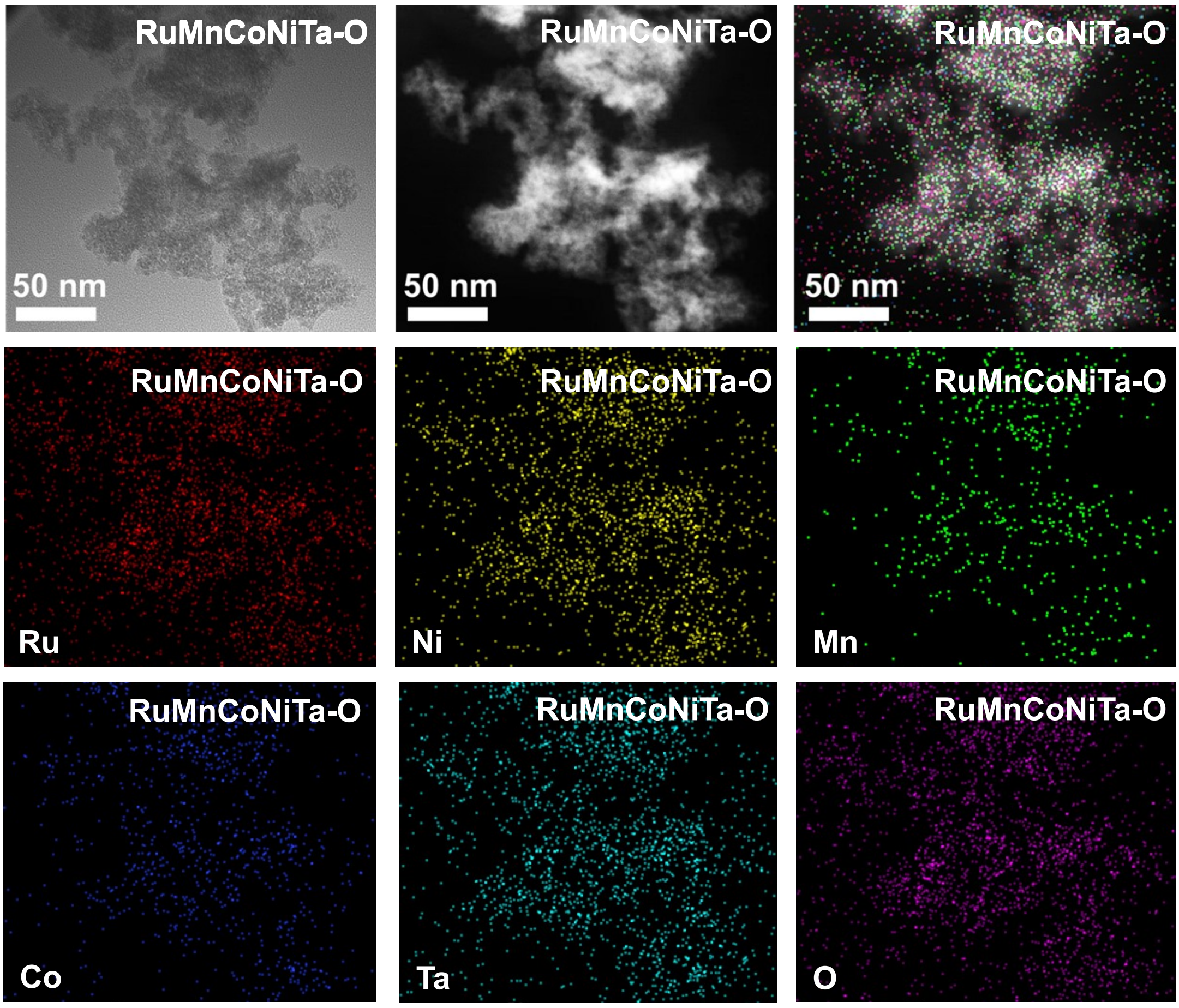

Figure S16. TEM images of RuMnCoNiTa-O and EDS of Ru, Mn, Co, Ni, Ta and O after long-term OER test.

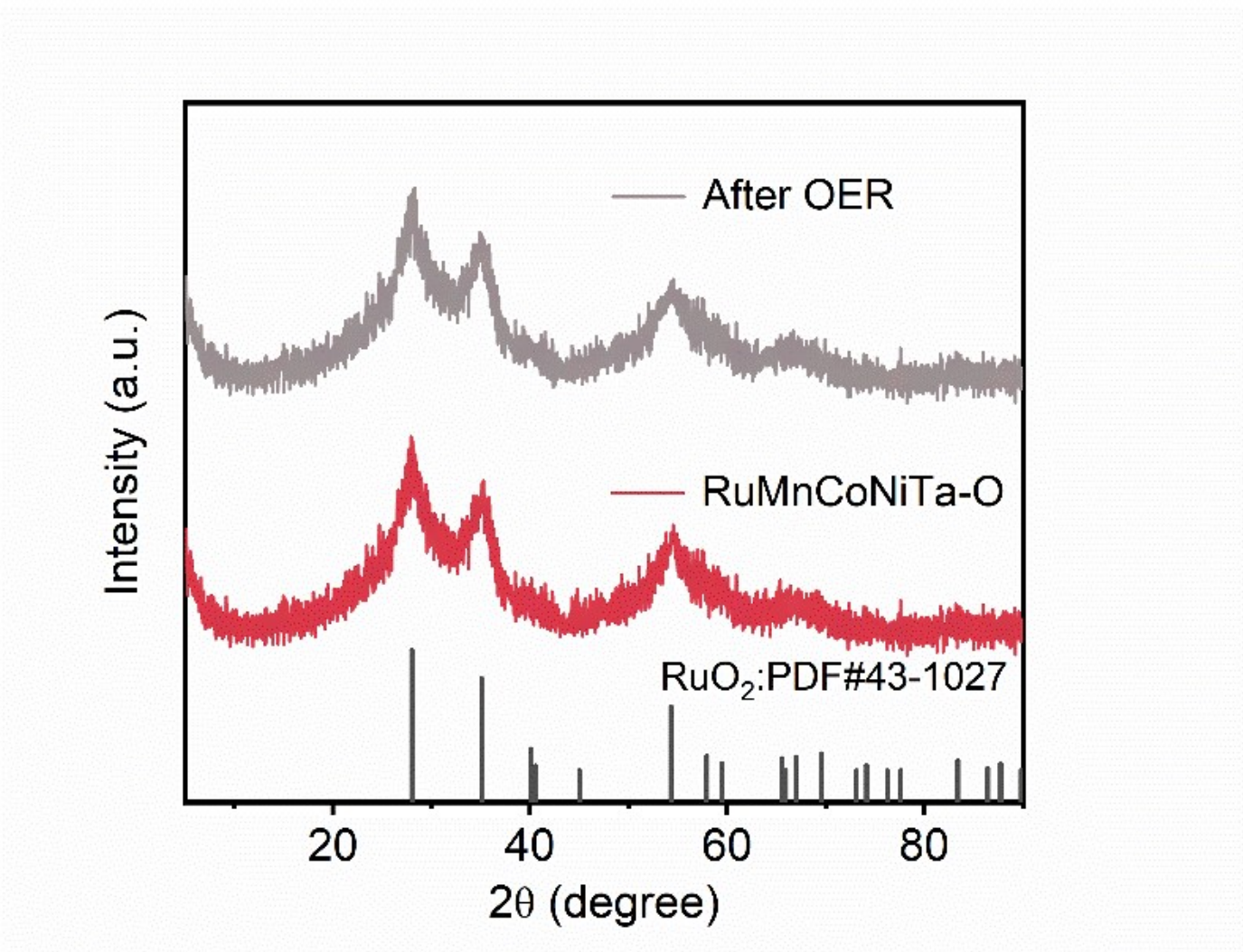

Figure S17. XRD patterns of RuMnCoNiTa-O before and after OER test.

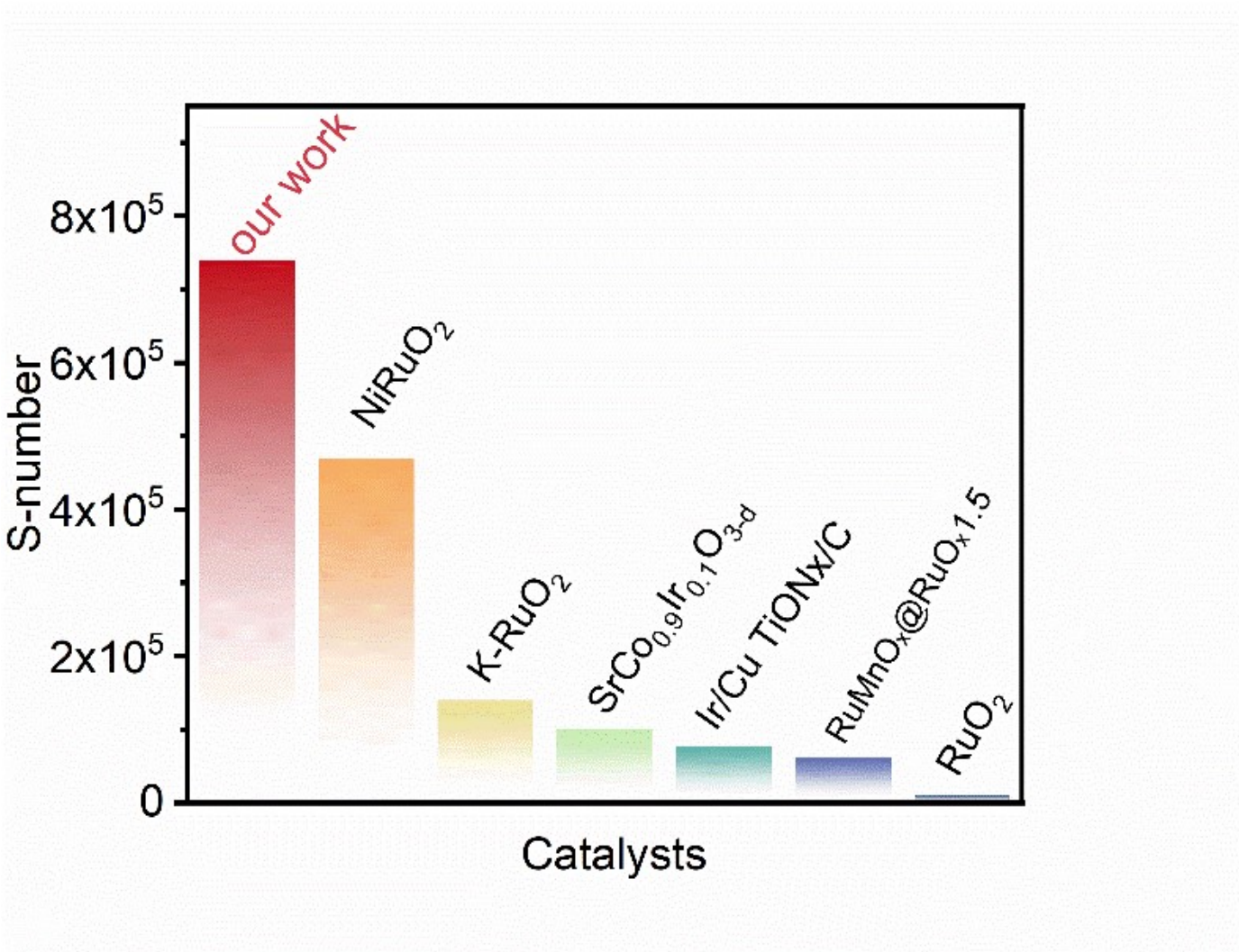

Figure S18. Comparison the S-number of RuMnCoNiTa-O with previously reported Ru/Ir-based electrocatalysts (**Table S3**).

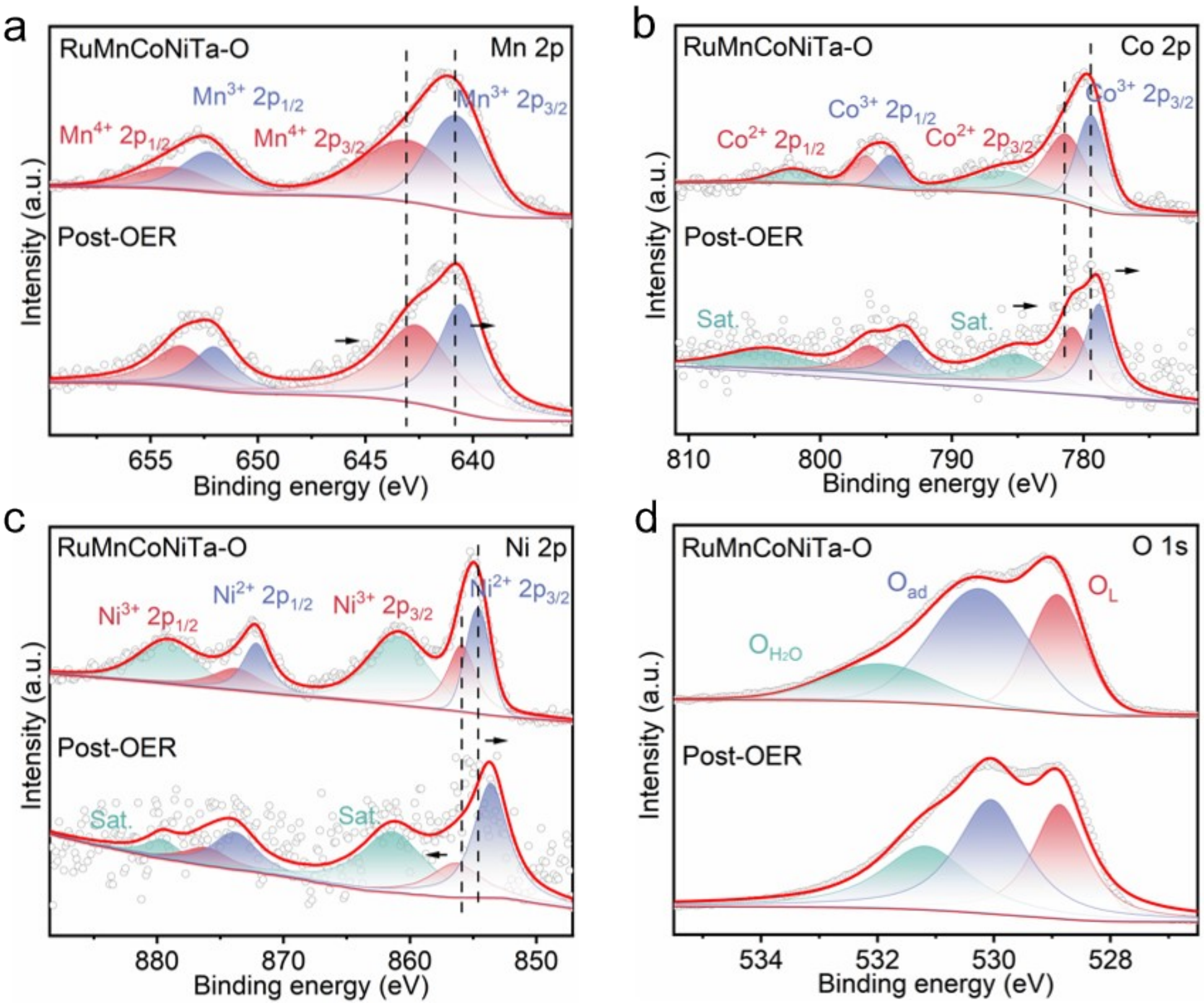

Figure S19. High-resolution XPS spectra of RuMnCoNiTa-O after OER test for (a) Mn 2p, (b) Co 2p, (c) Ni 2p, (d) O 1s.

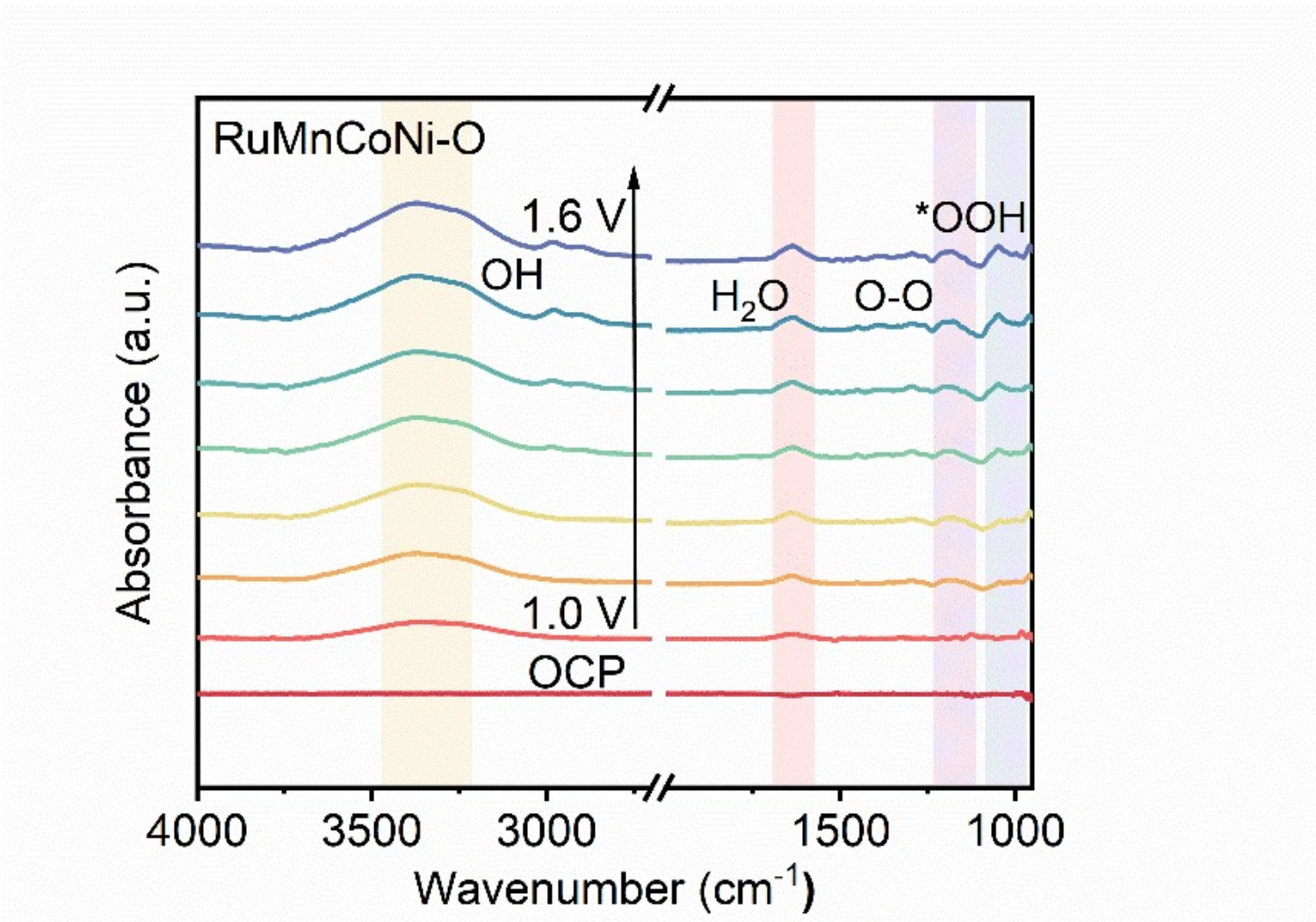

Figure S20. In-situ attenuated total reflection surface-enhanced infrared absorption spectroscopy (ATR-SEIRAS) of RuMnCoNi-O.

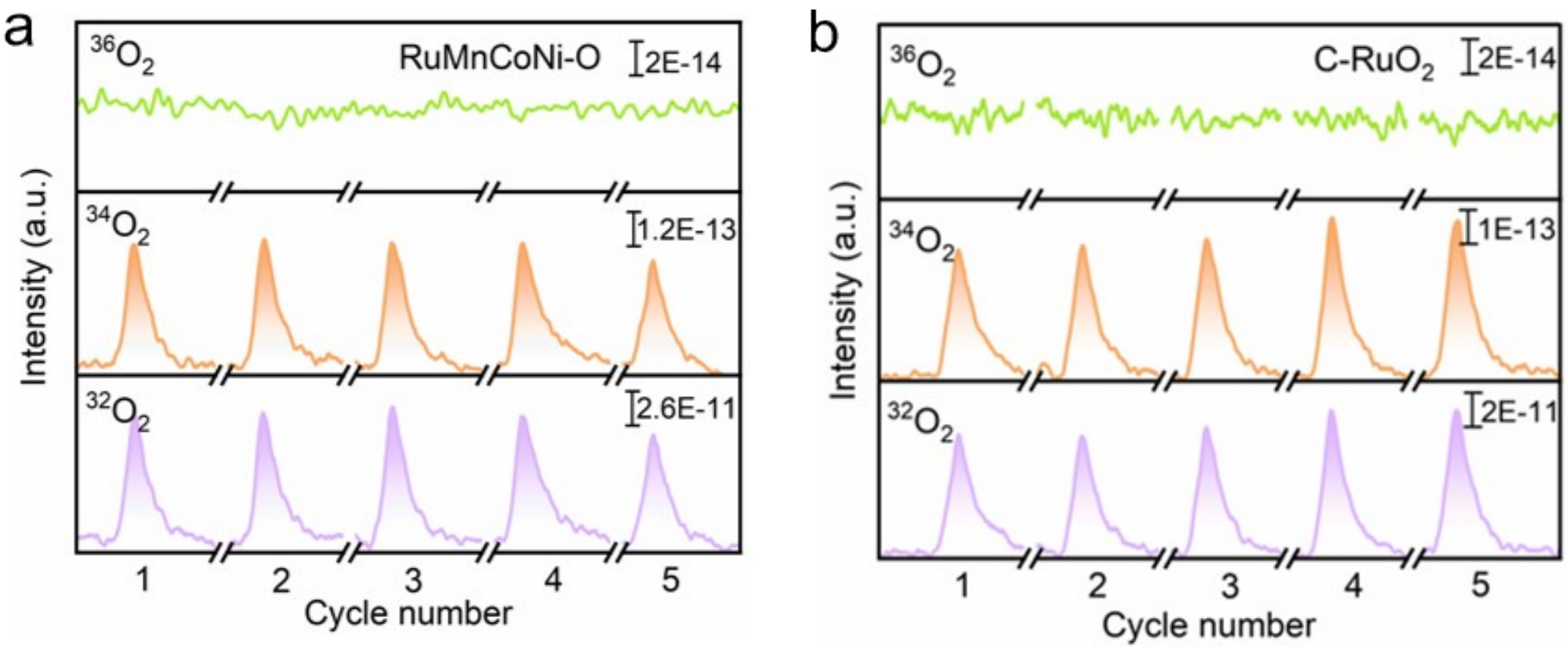

Figure S21. Online DEMS signals of gas products for O_2 for RuMnCoNi-O and C-RuO_2.

Prior to measurements, RuO_2 and RuMnCoNiTa-O are labeled with ^{18}O by five consecutive potential cycles in ^{18}O-enriched 0.5 M H_2SO_4, rinsed with pure water to remove residual $H_2{}^{18}O$, and then cycled in 0.5 M H_2SO_4 electrolyte. DEMS is employed to detect evolved O_2 species, namely $^{32}O_2$ ($^{16}O^{16}O$), $^{34}O_2$ ($^{16}O^{18}O$), and $^{36}O_2$ ($^{18}O^{18}O$), which originate from three distinct pathways: (i) two $H_2{}^{16}O$ molecules without lattice oxygen participation; (ii) one $H_2{}^{16}O$ molecule and one lattice ^{18}O; and (iii) two lattice oxygen atoms exclusively.

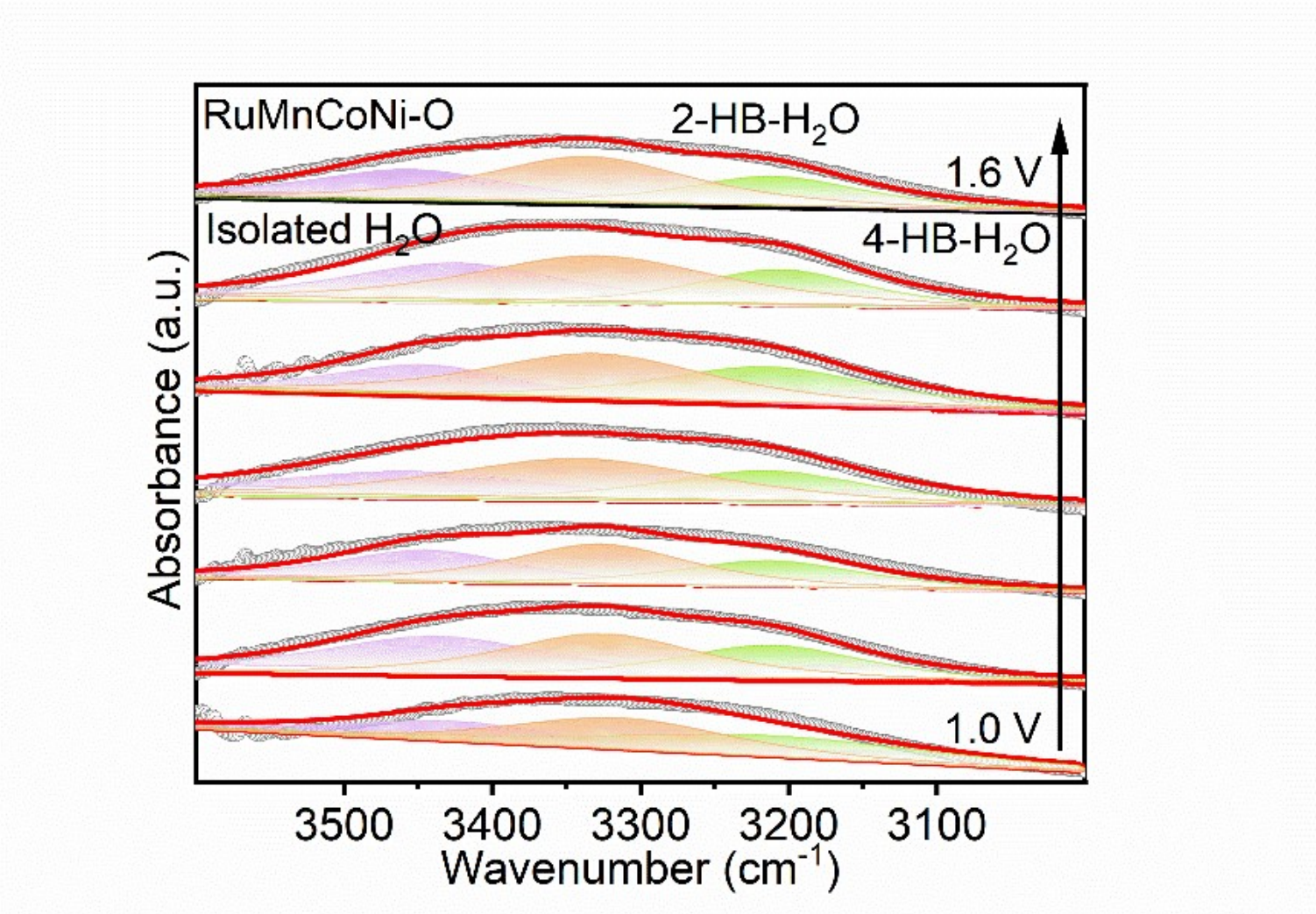

Figure S22. ATR-SEIRAS curves and deconvolution of O-H stretching peaks for RuMnCoNi-O.

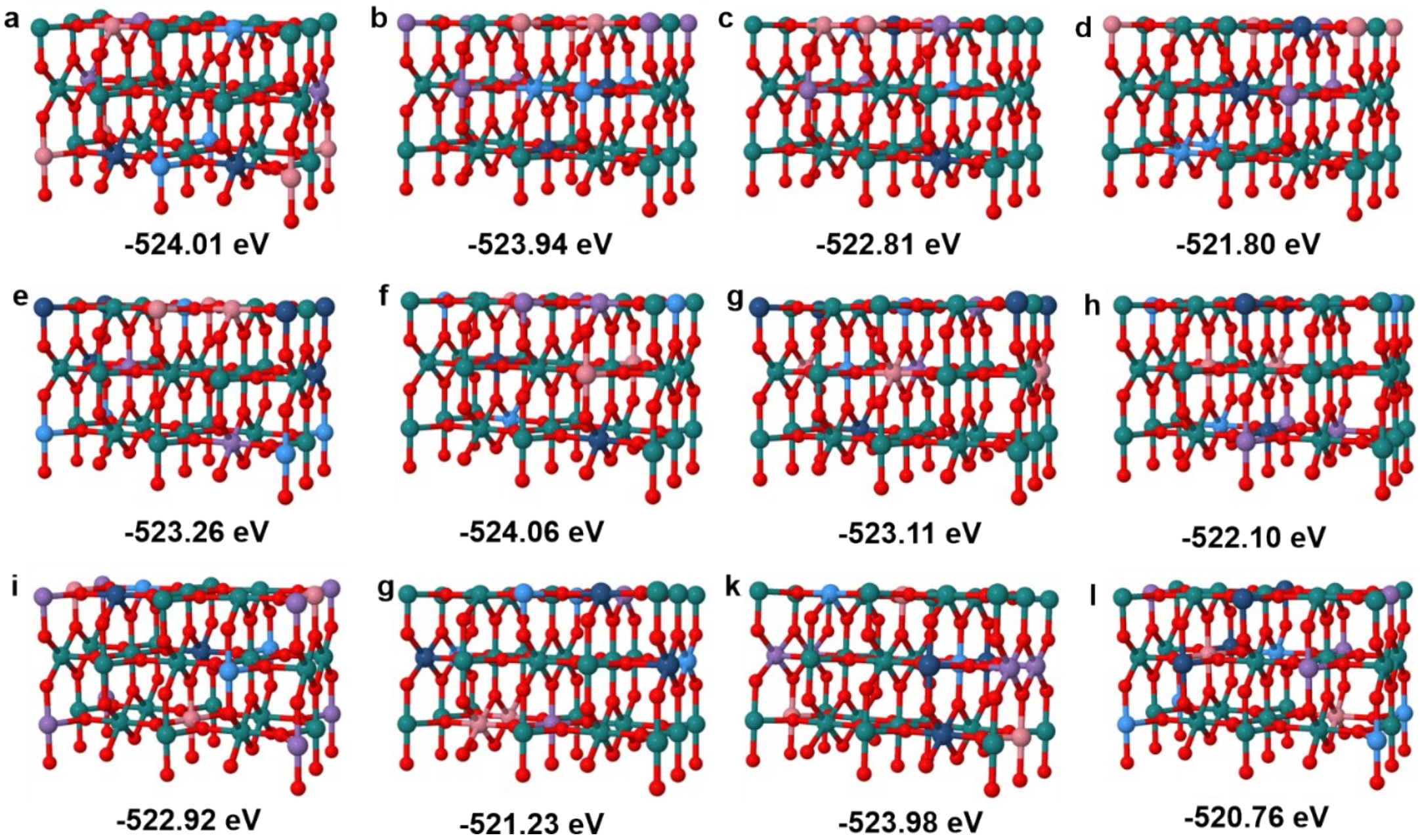

Figure S23. Twelve random configurations are screened by total formation energy.

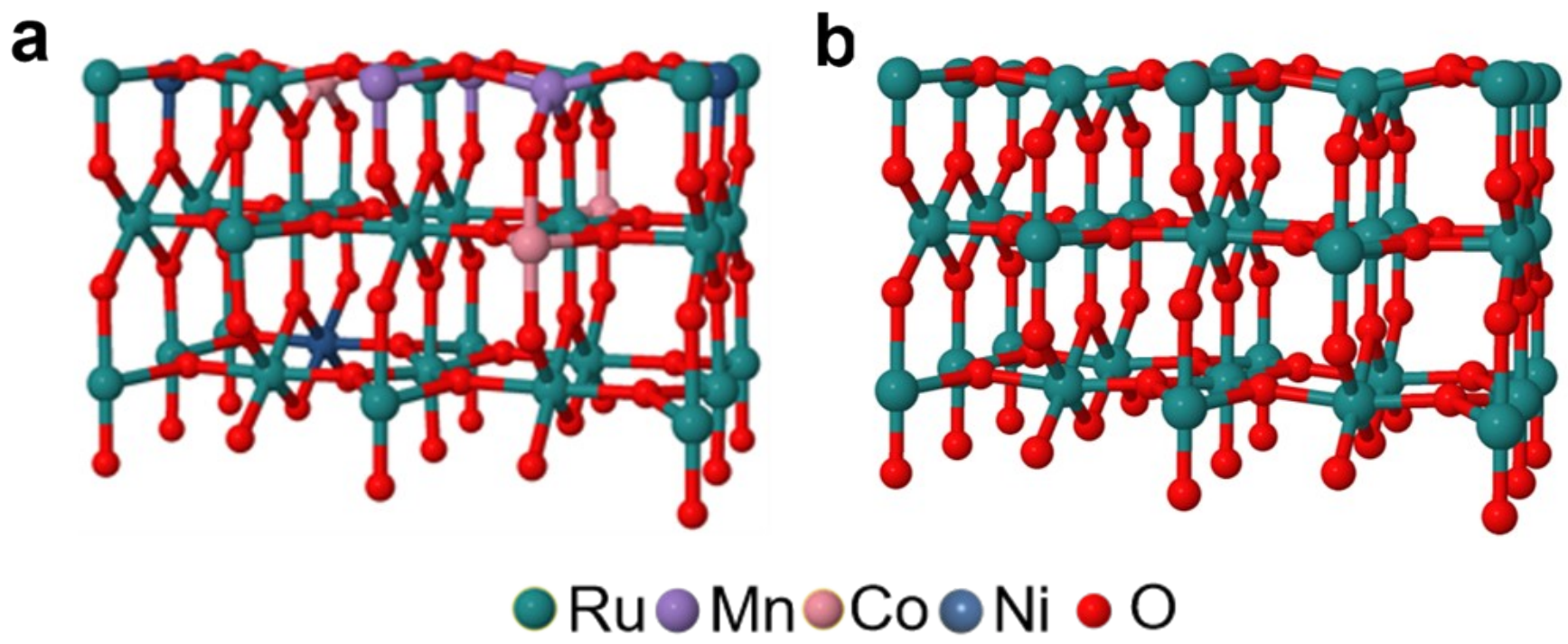

Figure S24. Optimized atomic structure models of RuMnCoNi-O and RuO_2.

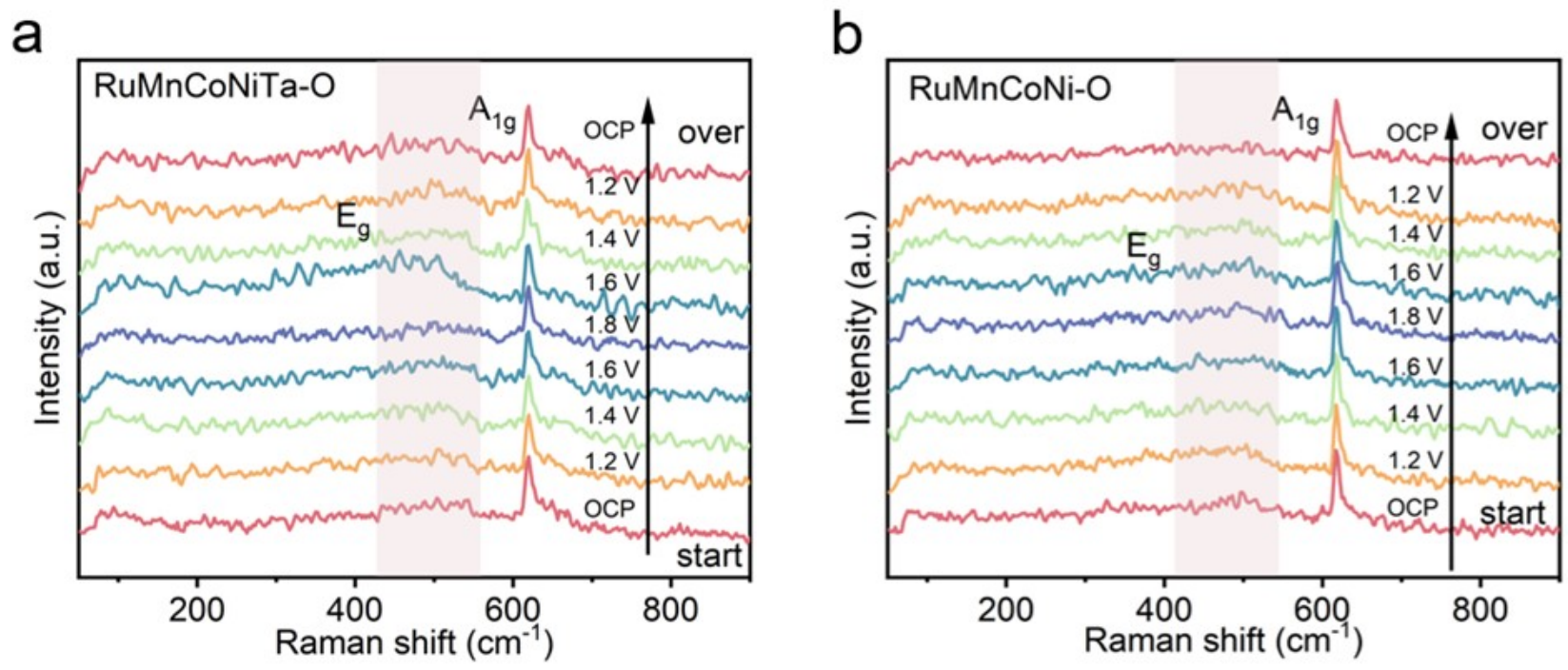

Figure S25. In situ Raman spectra obtained under various applied potential on (a) RuMnCoNiTa-O and (b) RuMnCoNi-O.

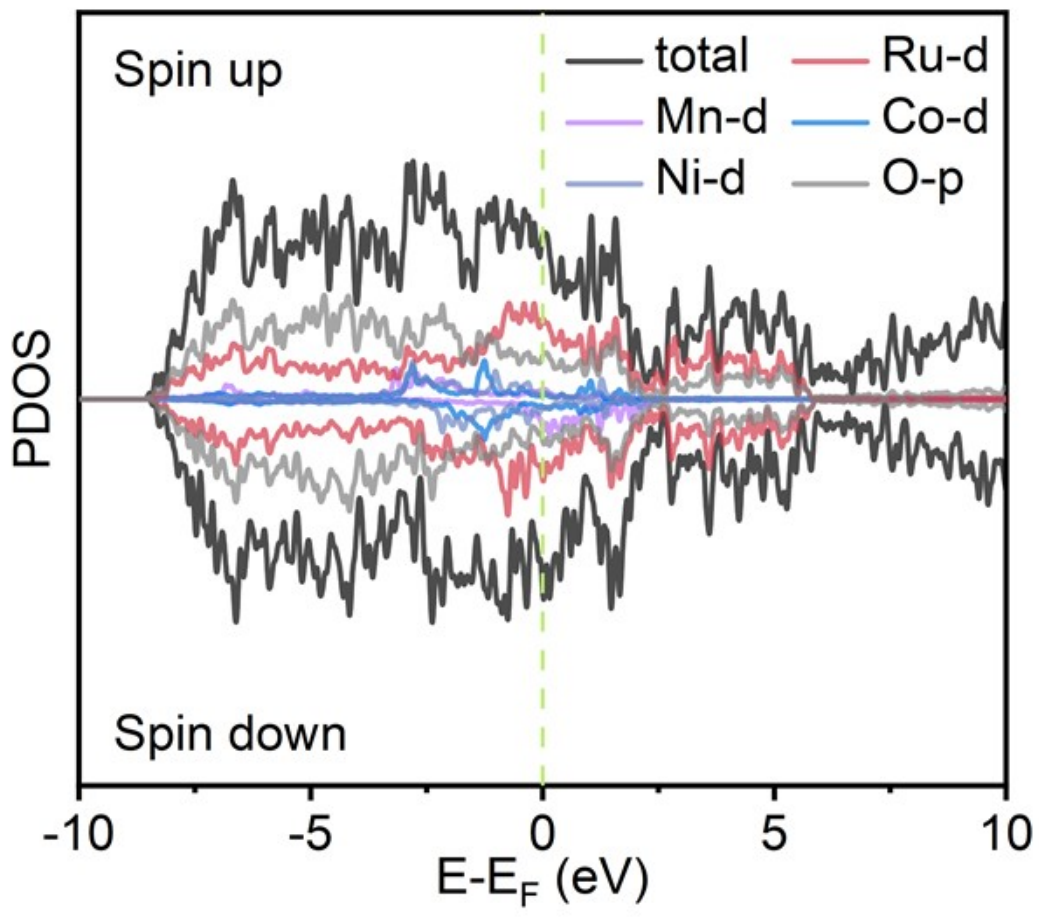

Figure S26. Projected density of states (PDOS) for Ru d-orbitals and O p-orbitals of RuMnCoNi-O.

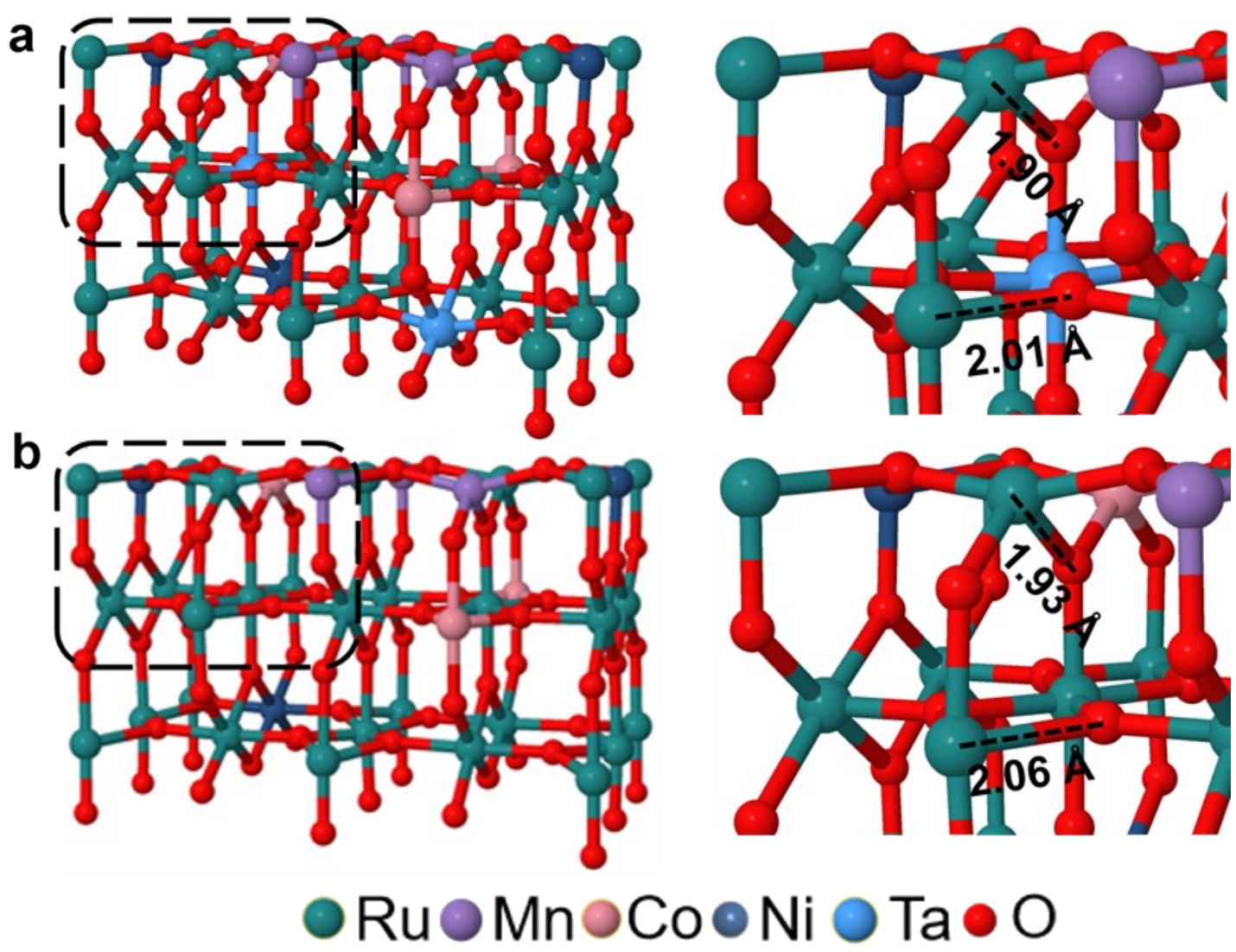

Figure S27. Optimized atomic configurations and calculated local Ru-O bond lengths for (a) RuMnCoNiTa-O and (b) RuMnCoNi-O high-entropy oxide framework.

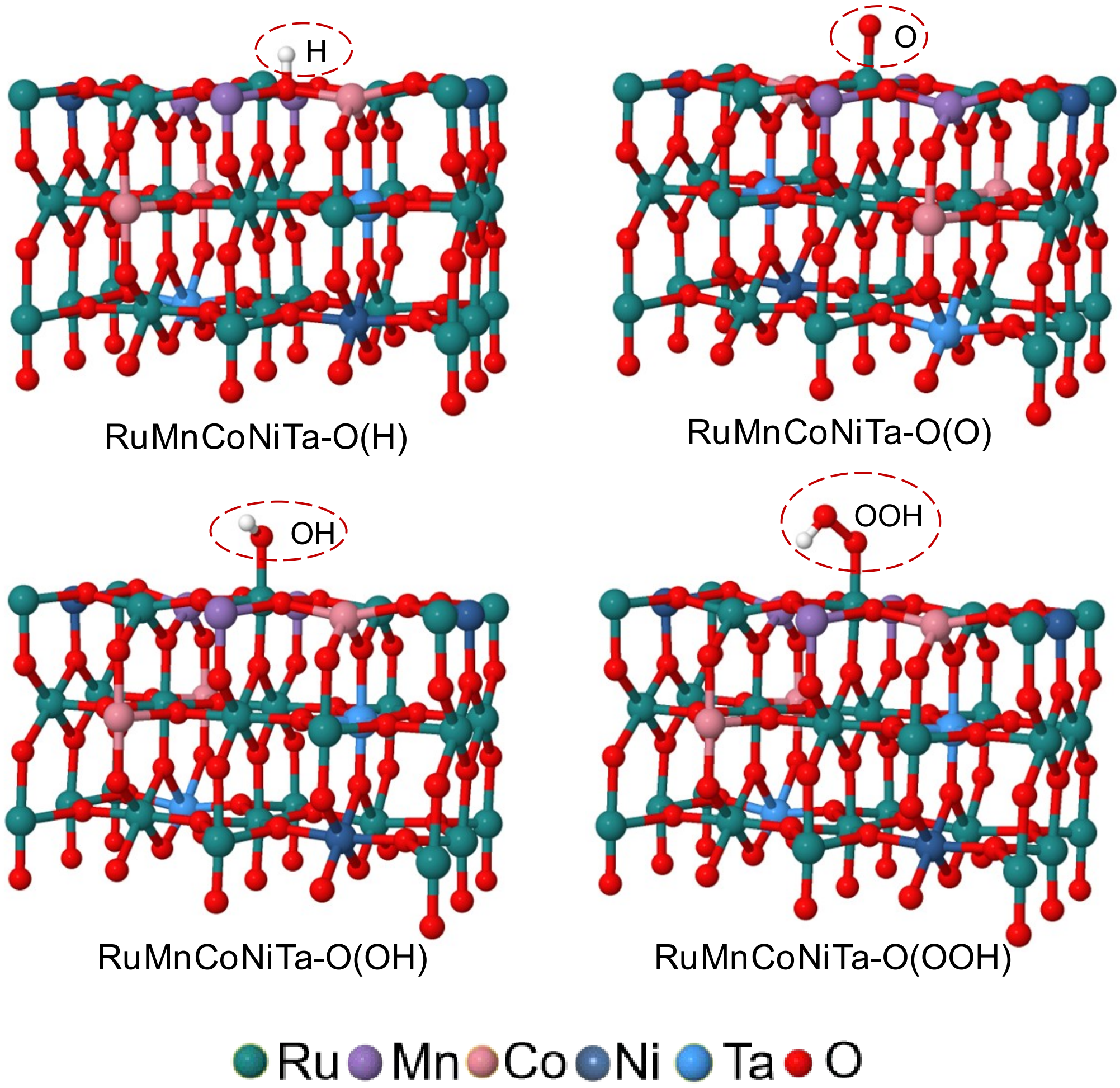

Figure S28. Schematic of adsorption steps for the AEM pathway in RuMnCoNiTa-O.

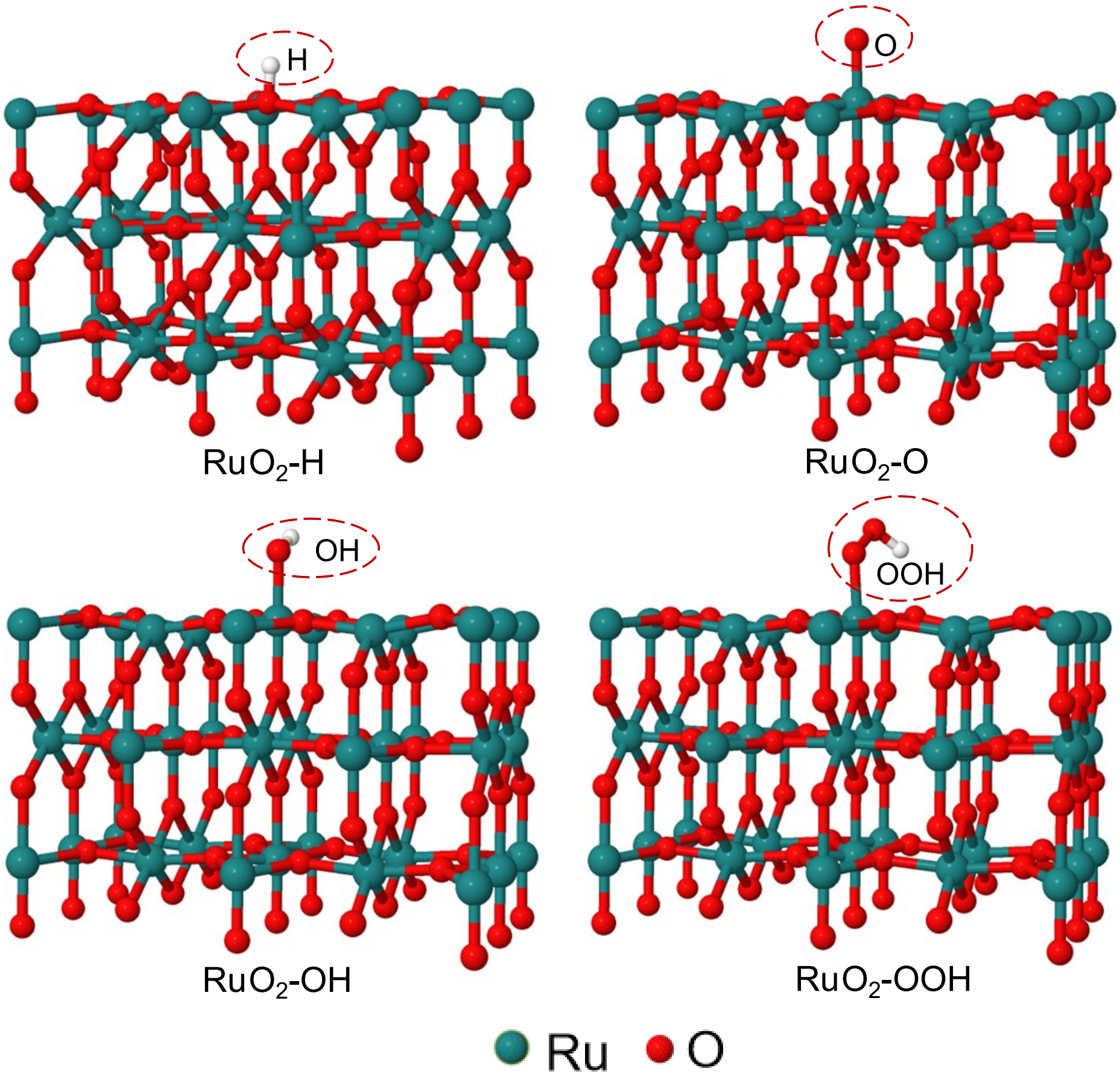

Figure S29. Schematic of adsorption steps for the AEM pathway in RuO_2.

Supporting Tables

Table S1. Fitting results of Ru K-edge EXAFS of RuMnCoNiTa-O, RuMnCoNi-O, Ta-RuO_2 and C-RuO_2.

Sample	Shell	N[a]	R(Å)[b]	σ^2(Å^2)[c]	ΔE_0(eV)[d]	R factor
C-RuO_2	Ru-O	5.771	1.982	0.002	3.224	0.020
Ru-foil	Ru-Ru	11.297	2.673	0.002	8.371	0.013
RuMnCoNi-O	Ru-O	5.253	1.965	0.003	3.616	0.014
RuMnCoNiTa-O	Ru-O	5.024	1.963	0.003	4.243	0.013

Table S2. Quantitative post-test dissolution analysis of all constituent metals in the electrolyte after 500 h of OER.

Element	concentration in electrolyte (mg L^{-1})	total mass loss relative to initial load (%)
Ru	0.37	1.86
Mn	0.0223	0.73
Co	0.028	0.87
Ni	0.0237	0.81
Ta	0.0201	0.68

Table S3. Comparison the S-number of RuMnCoNiTa-O with previously reported Ru/Ir-based electrocatalysts.

Electrocatalysts	S-number	References
RuMnCoNiTa-O	739720	**This work**
$NiRuO_2$	470000	*Nat. Chem.* **2023**, *22*, 100-108.
K-RuO_2	140000	*Chem. Eng. J.* **2023**, *454*, 140245.
$SrCo_{0.9}Ir_{0.1}O_{3-d}$	100000	*Nat. Commun.* **2019**, *10*, 572.
Ir/$CuTiON_x$/C	78000	*ACS Catal.* **2021**, *11*, 12510-12519.
$RuMnO_x$@RuO_x-1.5	62200	*ACS Catal.* **2024**, *14*, 12970-12981.
RuO_2	11297	*J. Am. Chem. Soc.* **2023**, *145*, 17995-18006.

Table S4. Summary of recently reported Ru-based catalysts for acidic water oxidation.

Catalysts	Overpotential @10 mA cm^{-2}(mV)	Durability (h)	Reference
RuMnCoNiTaO	196	508	**This work**
$Ru_{0.6}W_{17.4}O_{49-\delta}$	252	45	[6]
$Pr_3Ir_{1-x}Mo_xO_7$	259	200	[7]
IrO_2@TaB_2	288	120	[8]
RuO_2-WC	347	10	[9]
RuO_2/(Co, Mn)$_3O_4$	270	24	[10]
$Cr_{0.6}Ru_{0.4}O_2$	178	10	[11]
HP-RuO_x	237	140	[12]
COF-205-Ru	212	280	[13]
S_H-RuCuO	231	250	[14]
Ru/S NSs-400	219	600	[15]
Ga-RuO_2	217.5	150	[16]
$Li_{0.52}RuO_2$	156	70	[17]
IrRuNiMoCo	243	24	[18]
HEA/PANI-CP	258	300	[19]
SS Pt-RuO_2	228	100	[20]
a/c-RuO_2	220	60	[21]
Co-Ru@RuO_2 9:1	203	420	[22]
Mn/Ru-Co_3O_4	230	120	[23]
RuO_2/0.5CeO_2	214	550	[24]
(RuNiFeMoCr)$_3O_4$	236	450	[25]
RuMnO_x@RuO_x-1.5	192	240	[26]

Table S5. Calculated total energies (E_{total}), energy differences (ΔE), and intermediate adsorption energies (Eads) for *OH adsorption on different metal sites of RuMnCoNiTa-O.

Adsorption Site	E_{total} (eV)	ΔE (eV)	E_{ads} Adsorption Energy (eV)
Ru	-542.785	-10.874	-0.039
Mn	-542.591	-10.680	+0.155
Co	-541.960	-10.049	+0.786
Ni	-543.018	-11.111	-0.272
Ta	-543.014	-11.103	-0.268

Reference

[1] G. Kresse, J. Furthmüller, Efficiency of Ab-initio total energy calculations for metals and semiconductors using a plane-wave basis set, Comput. Mater. Sci. 6 (1996) 15-50, https://doi.org/10.1016/0927-0256(96)00008-0.

[2] G. Kresse, J. Furthmüller, Efficient iterative schemes for ab initio total-energy calculations using a plane-wave basis set, Phys. Rev. B 54 (1996) 11169-11186, https://doi.org/10.1103/PhysRevB.54.11169.

[3] J. P. Perdew, K. Burke, M. Ernzerhof, Generalized gradient approximation made simple, Phys. Rev. Lett. 78 (1997) 1396, https://doi.org/10.1103/PhysRevLett.78.1396.

[4] H. J. Monkhorst, J. D. Pack, Special points for brillouin-zone integrations, Phys. Rev. B 13 (1976) 5188-5192, https://doi.org/10.1103/PhysRevB.13.5188.

[5] M. Bajdich, M. García-Mota, A. Vojvodic, J. K. Nørskov, A. T. Bell, Theoretical investigation of the activity of cobalt oxides for the electrochemical oxidation of water, J. Am. Chem. Soc. 135 (2013) 13521-13530, https://doi.org/10.1021/ja405997s.

[6] X. Wang, H. Jang, S. Liu, Z. Li, X. Zhao, Y. Chen, M. G. Kim, Q. Qin, X. Liu, Enhancing the catalytic kinetics and stability of Ru sites for acidic water oxidation by forming brønsted acid sites in tungsten oxide matrix, Adv. Energy Mater. 13 (2023) 2301673, https://doi.org/10.1002/aenm.202301673.

[7] S. Chen, S. Zhang, L. Guo, Lun Pan, C. Shi, X. Zhang, Z. Huang, G. Yang, J. Zou, Reconstructed Ir-O-Mo species with strong brønsted acidity for acidic water oxidation, Nat. Commun. 14 (2023) 4127, https://doi.org/10.1038/s41467-023-39822-6.

[8] Y. Wang, M. Zhang, Z. Kang, L. Shi, Y. Shen, B. Tian, Y. Zou, H. Chen, X. Zou, Nano-metal diborides-supported anode catalyst with strongly coupled TaO_x/IrO_2 catalytic layer for low-iridium-loading proton exchange membrane electrolyzer, Nat. Commun. 14 (2023) 5119, https://doi.org/10.1038/s41467-023-40912-8.

[9] S. Sun, H. Jiang, Z. Chen, Q. Chen, M. Ma, L. Zhen, B. Song, C. Xu, Bifunctional WC-supported RuO_2 nanoparticles for robust water splitting in acidic media, Angew. Chem. Int. Ed. 61 (2022) e202202519, https://doi.org/10.1002/anie.202202519.

[10] S. Niu, X. Kong, S. Lia, Y. Zhang, J. Wu, W. Zhao, P. Xu, Low Ru loading $RuO_2/(Co,Mn)_3O_4$ nanocomposite with modulated electronic structure for efficient oxygen evolution reaction in acid, App. Catal. B: Environ. 297 (2021) 120442, https://doi.org/10.1016/j.apcatb.2021.120442.

[11] Y. Lin, Z. Tian, L. Zhang, J. Ma, Z. Jiang, B. J Deibert, R. Ge, L. Chen, Chromium-ruthenium oxide solid solution electrocatalyst for highly efficient oxygen evolution reaction in acidic media, Nat. Commun. 10 (2019) 162, https://doi.org/10.1038/s41467-018-08144-3.

[12] J. Wang, L. Cai, Z. Yu, H. Tan, X. Xiang, K. Xu, Y. Chao, S. M. Thalluri, F. Lin, H. Huang, C. Zhang, Y. Zhao, W. Wang, L. Liu, Oxygen-defective ruthenium oxide as an efficient and durable electrocatalyst for acidic oxygen evolution reaction, J. Mater. Chem. A 13 (2025) 312-324, https://doi.org/10.1039/D4TA06592A.

[13] H. Jia, N. Yao, Y. Jin, L. Wu, J. Zhu, W. Luo, Stabilizing atomic Ru species in conjugated sp2 carbon-linked covalent organic framework for acidic water oxidation, Nat. Commun. 15 (2024) 5419, https://doi.org/10.1038/s41467-024-49834-5.

[14] Q. Yao, Z. Yu, Y. Chu, Y. Lai, T. Chan, Y. Xu, Q. Shao, X. Huang, S incorporated RuO_2-based nanorings for active and stable water oxidation in acid, Nano Res. 15 (2022) 3964-3970, https://doi.org/10.1007/s12274-022-4081-4.

[15] L. Liu, Y. Ji, W. You, S. Liu, Q. Shao, Q. Kong, Z. Hu, H. Tao, L. Bu, X. Huang, Trace lattice S inserted RuO_2 flexible nanosheets for efficient and long-term acidic oxygen evolution catalysis, Small 19 (2023) 2208202, https://doi.org/10.1002/smll.202208202.

[16] L. Wu, W. Huang, D. Li, H. Jia, B. Zhao, J. Zhu, H. Zhou, W. Luo, Unveiling the structure

and dissociation of interfacial water on RuO_2 for efficient acidic oxygen evolution reaction, Angew. Chem. Int. Ed. 64 (2025) e202413334, https://doi.org/10.1002/anie.202413334.
[17] Y. Qin, T. Yu, S. Deng, X. Zhou D. Zhang,Y. He, H. Qiu, D. Lin, Q. Zhang, Z. Jin, L. He, F. Kang, K. Li, T. Zhang, RuO_2 electronic structure and lattice strain dual engineering for enhanced acidic oxygen evolution reaction performance, Nat. Commun. 13 (2022) 3784, https://doi.org/10.1038/s41467-022-31468-0.
[18] L. Yao, F. Zhang, S. Yang, S. Yang, H. Zhang, Y. Li, C. Yang, H. Yang, Q. Cheng, Sub-2 nm Ir Ru Ni Mo Co high-entropy alloy with Iridium-rich medium-entropy oxide shell to boost acidic oxygen evolution, Adv. Mater. 36 (2024) 2314049, https://doi.org/10.1002/adma.202314049.
[19] L. Su, X. Chen, Y. Tan, W. Deng, H. Wu, X. Miao, Suppressing migration of Ru in a high-entropy alloy for durable acidic oxygen evolution, J. Mater. Chem. A 13 (2025) 15743-15747, https://doi.org/10.1039/D5TA01949D.
[20] J. Wang, H. Yang, F. Li, L. Li, J. Wu, S. Liu, T. Cheng, Y. Xu, Q. Shao, X. Huang, Single-site Pt-doped RuO_2 hollow nanospheres with interstitial C for high-performance acidic overall water splitting, Sci. Adv. 8 (2022) eabl9271, https://doi.org/10.1126/sciadv.abl9271.
[21] L. Zhang, H. Jang, H. Liu, M. G. Kim, D. Yang, S. Liu, X. Liu, J. Cho, Sodium-decorated amorphous/crystalline RuO_2 with rich oxygen vacancies: a robust pH-universal oxygen evolution electrocatalyst, Angew. Chem. Int. Ed. 60 (2021) 18821, https://doi.org/10.1002/anie.202106631.
[22] J. Chen, Y. Ma, C. Cheng, T. Huang, R. Luo, J. Xu, X. Wang, T. Jiang, H. Liu, S. Liu, T. Huang, L. Zhang, W. Chen, Cobalt-doped Ru@RuO_2 core–shell heterostructure for efficient acidic water oxidation in low-Ru-loading proton exchange membrane water electrolyzers, J. Am. Chem. Soc. 147 (2025) 8720-8731, https://doi.org/10.1021/jacs.4c18238.
[23] Q. Fu, L. W. Wong, X. Zheng, C. S. Tsang, H. Chen, W. Shen, T. Ling, T. H. Ly, Q. Deng, J. Zhao, The role of oxygen vacancies: Triggering lattice oxygen oxidation mechanism in acidic OER, Nano Energy 149 (2026) 111731, https://doi.org/10.1016/j.nanoen.2026.111731.
[24] T. Liu, Q. Zhou, L. Yang, Y. Zhang, R. Wang, Z. Chen, Rational design of a RuO_2/0.5CeO_2 heterostructure as an efficient and stable electrocatalyst for acidic water splitting, J. Mater. Chem. A 14 (2026) 26036-26045, https://doi.org/10.1039/d6ta01093h.
[25] Y. Tu, X. Shan, L. Chen, J. Zhang, L. Yang, F. Cai, C. Zhong, P. Wang, H. Gao, J. Xu, Z. Jin, B. Cai, Z. Cui, M. Zhou, Accelerated discovery of highly stable ruthenium-based high-entropy oxides for acidic oxygen evolution, Sci. Adv. 12 (2026) eaed8479, https://doi.org/10.1126/sciadv.aed8479.
[26] Y. Qin, X. Niu, R. Zhao, J. Sun, Z. Xu, Z. Guo, D. Liu, L. Guo, C. Liu, J. Zhang, Q. Wang, Manganese as electron reservoir stabilized $RuMnO_x$@RuO_x with enhanced activity and robust durability for acidic water oxidation, ACS Catal. 14 (2024) 12970-12981, https://doi.org/10.1021/acscatal.4c01707.
